# Wireless power and information dual transfer system via magnetically coupled resonators

**DOI:** 10.1038/s44172-023-00154-4

**Published:** 2024-01-08

**Authors:** Xiangning He, Sheng Liu, Jiande Wu, Yue Feng, Ruichi Wang, Wuhua Li, Wanying Weng

**Affiliations:** 1 College of Electrical Engineering, Zhejiang University, 310027 Hangzhou, Zhejiang China; 2 School of Mechanical, Electrical and Manufacturing Engineering, Loughborough University, Loughborough, Leicestershire LE11 3TU UK

**Keywords:** Electrical and electronic engineering, Devices for energy harvesting

## Abstract

High-efficiency medium-range wireless power transfer using magnetically coupled resonators requires a wireless data link between the contactless coils to regulate power. Multiplexing the power transfer channel as the information channel is a cost-effective solution for the communication. However, existing technologies cannot transmit data across the medium-range magnetically coupled resonators channel without substantially affecting power transfer. Here we show a power-electronics-converters based wireless power and information dual transfer system in which the information signals are modulated on one dc side of the inverter/rectifier, and transmitted through a conventional medium-range wireless power transfer system, and then demodulated on the other dc side. Using the frequency mixer characteristic of the inverter/rectifier, information is modulated onto the sideband of the power carrier and transmitted through the medium-range channel. Finally, we prototyped a 6.78 MHz system capable of transferring 45 W power across a one-meter distance with 62% efficiency and 60 kb/s bitrate for half-duplex communication.

## Abstract (web summary)

Medium-range wireless power transfer using magnetically coupled resonators requires a wireless data link between the contactless coils to regulate power. Here, Xiangning He and colleagues report a wireless power transfer system which efficiently multiplexes the information modulation and signal channel with the power conversion and transfer channel.

## Introduction

Wireless power transfer (WPT) is a technology that transfers electrical power over distances without interconnecting wires. Compared to wired electricity transmission, the WPT technology can bring several important advantages such as increasing the mobility, convenience, and safety of electronic devices for all users^1,2^. In 1904, the WPT concept was first proposed by Nikola Tesla to develop a global system with “transmission of electrical energy without wires”^3^. Since then, WPT had long attracted popular curiosity and scientific interest. Recently, the need for WPT surges due to the growing popularity of mobile electrical products. To meet the requirement of fast charging for mobile phones, the output power typically needs to be at least 10 W, as specified by Qualcomm quick charge 1.0 standard^4^.

Over the years, different WPT technologies have been investigated and implemented. Inductive power transfer (IPT) is a proven short-range WPT technology that has been widely used in wireless charging systems for mobile phones and electrical cars^5,6^. However, the power transmission distance of IPT systems is usually limited to a few centimeters, which diminishes the benefit and restricts the application of IPT technology. On the other side, the near field communication with powering capability technologies, such as NFC, is generally incapable of delivering power larger than 0.5 W, and the distance for power transfer is typically less than 0.1 m. It cannot be used for applications such as quick charging for mobile phones requiring at least 10 W power.

**Fig. 1:**
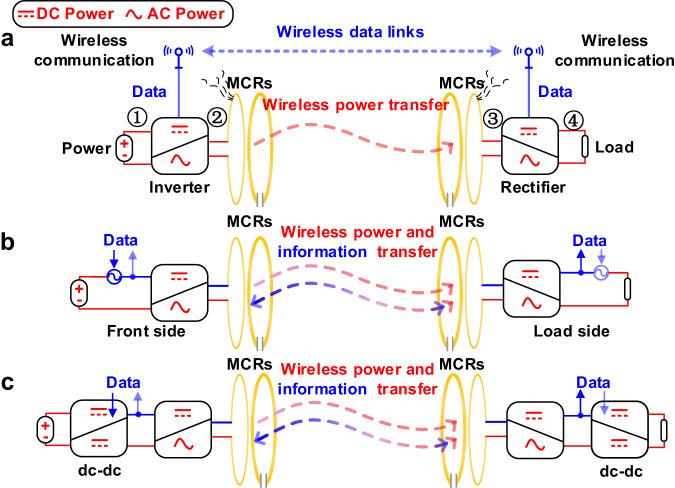
Schematic diagram of the MCR-WPT system. **a** With an independent data link. **b** The original concept of WPIDT technology. **c** A practical WPIDT scheme by multiplexing cascaded dc-dc regulators for data transmitter. Red line denotes power flow path and blue line denotes data transmission path.

The WPT technology using magnetically coupled resonators (MCR) is a promising technology that extends the distances of power transfer while maintaining high efficiency^7^. In general, an MCR-based WPT (MCR-WPT) system operate at megahertz frequencies (such as 6.78 and 13.56 MHz) and can provide dozens of watts to portable devices more than one-meter away^7–11^. A basic MCR-WPT system driven by power electronics converters (Fig. 1a) typically consists of an inverter on the front side, a rectifier on the load side, and a pair of coil-based resonators. In operation, the dc power ① on the front side is first converted to ac power ②, then, transmitted to the load side through MCR ③, and finally rectified to dc power for the load ④. Power is delivered wirelessly, and the output variables, such as voltage and current, must be fed back to the front side for proper control. Therefore, a reliable data link between the front and load sides is required. Wireless communication, such as Wi-fi, Bluetooth, and Zigbee, is a potential candidate technology for MCR-WPT (Fig. 1a). However, adding extra wireless communication devices to an MCR-WPT system increases not only the cost but also the risk of spatial interference^12^. To ensure communication security and proper pairing, it is highly desirable that data transfer should be tightly coupled with power transfer in an MCR-WPT system.

The technologies that transmit power and information simultaneously are generally referred to as simultaneous wireless power and information/data transfer (SWPIT/SWPDT) technology in high power applications (typically greater than several watts) and as simultaneous wireless information and power transfer (SWIPT) technology in low power applications. Although these technologies have been extensively studied in recent years, it remains challenging to communicate with high power (typically>10 W) over a medium-range MCR channel (typically>50 cm) while maintaining high power transfer efficiency (typically>50%). In a duplex communication system with these features, the upward communication, i.e. information from the load to the power transmitter, presently has a greater challenge compared to the download communication. Load shift keying (LSK) modulation is a common method for upward transmission in IPT devices compliant with the Qi Standard^13^, but it would have a substantial impact on the power transfer efficiency in high power applications. On the other hand, in high power SWPIT systems, the methods that multiplex the power coils to transmit data signals with a different frequency carrier^14–18^, are not applicable to an MCR-WPT system over long distance, because MCR would block signals that does not fall within its passband^19^.

The power electronics and communication technology share fundamental principles and common circuit topologies^20–22^, there are chances for us to perform inter-disciplinary research^23,24^. For example, a full bridge circuit in power electronics is either an inverter or a rectifier, whilst in communication systems, it is a frequency mixer that modulates a signal from one frequency to another. Indeed, the inverter and rectifier can be modelled as the product of a dc or ac input signal multiplied by a square wave of a specific frequency, exactly as the frequency mixer. Furthermore, we observed that the front side inverter and the load side rectifier in an MCR-WPT system are jointed to be a pair of frequency mixers with synchronous local oscillators. The synchronous nature of the inverter and rectifier enables the data signal with a low frequency carrier to commute between the two sides of the system, thereby establishing reliable and efficiently wireless data links between them, as shown in Fig. 1b. Thanks to the synchronous nature of the inverter and rectifier, we propose a wireless power and information dual transfer (WPIDT) system, in which the power and information are multiplexed on the dc side, and transmitted through a conventional MCR-WPT system, and then de-multiplexed on the other dc side.

In the WPIDT system, the information source may be introduced through a specific signal coupling circuit or by a cascaded dc-dc converter. As shown in Fig. 1c, conventional MCR-WPT system usually employs two cascaded dc-dc converters, one on the front side to regulate output power and the other on the load side to maintain the rectifier’s output impedance at an optimal value to maximize power transfer efficiency. Despite the additional small power losses introduced by the dc-dc stage, the system can effectively maintain a high level of efficiency in applications with variable distance and variable power^25,26^. In the WPIDT scheme, both dc-dc converters can also be multiplexed as signal sources for upward and downward transmission, respectively. Thus, each power electronics converter in Fig. 1c is multiplexed as a communication system component. The power and data are simultaneously modulated and transmitted, providing the cross-check between power transfer and communication, which ensures the essential security and robustness of the system. To demonstrate the performance and advantages of the design, we developed a 6.78 MHz MCR-WPT system capable of efficiently transferring 45 W of power across a one-meter distance with high efficiency. A bit rate of 60 kb/s for half-duplex communication has been achieved, proving the effectiveness of the scheme.

The proposed WPIDT system changes the power-only MCR-WPT system to an integrated power and information transmission system, and offers a cost-effective solution for the medium-range simultaneous transmission of information based on power electronics converters in MCR-WPT systems. The system has important advantages in power and information transmission cost, distance, efficiency, and security, and expected to be widely applied in power electronics devices.

## Results

### Synchronous nature of inverter and rectifier in MCR-WPT system

**Fig. 2:**
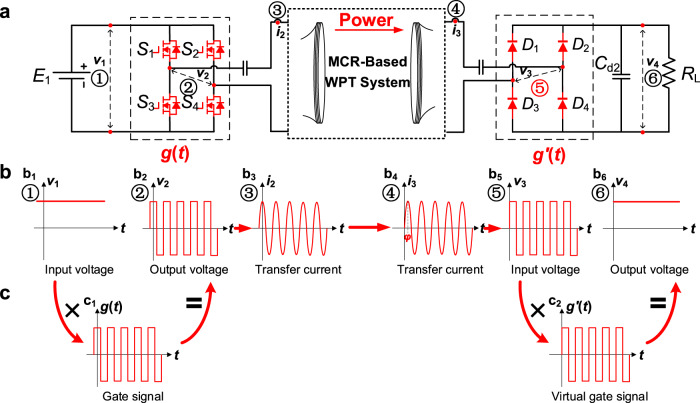
Waveforms of power flow in MCR-WPT system. **a** Circuit diagram of the MCR-WPT system. *S*_1_ ~ *S*_4_ denote the switches of the inverter and *D*_1_ ~ *D*_4_ denote the diodes of the rectifier. *E*_1_ is the input dc source, *C*_d2_ is the filter capacitance and *R*_L_ is the load resistance. **b** Waveforms at position ①-⑥ and their relationship. b_1_ is the input dc voltage *v*_1_ at ①, b_2_ is the mixed output voltage *v*_2_ at ②, b_3_ is the front side transfer current *i*_2_ at ③, b_4_ is the load side current *i*_3_ at ④, b_5_ is the load side input voltage *v*_3_ at ⑤, and b_6_ is the mixed output dc voltage *v*_4_ at ⑥. *φ* is the phase of the current and red line denotes the power transfer waveforms. **c** Waveforms of the local oscillator *g*(*t*) and *g*^′^(*t*).

In MCR-WPT system, the magnetic resonance between the coupled coil resonators is the key to efficient wireless power transfer, and only electric waves with the same frequency as the resonance may pass through the MCR channel. Fig. 2a depicts a typical MCR-WPT system based on power electronic converters. On the front side, the full-bridge inverter outputs a square wave (Fig. 2b2). Assuming that the switching frequency of the inverter is equal to the resonant frequency *f*_0_, only the fundamental component of the square wave can pass through the MCR network, resulting in a sinusoidal current wave (Fig. 2b3). In this process, the inverter is controlled by active switches such as power MOSFET, and the mathematical expression of the converter is

1 $${v}_{2}={E}_{1}\cdot g(t)$$

where ${E}_{1}$, ${v}_{2}$ are the input and output voltages of the inverter, and ${g}(t)$ is a unit square wave function, defined as

2 $${g}(t)=\left\{\begin{array}{c}1,\frac{1}{{f}_{0}}\left(n-\frac{1}{4}\right)\le t < \frac{1}{{f}_{0}}\left(n+\frac{1}{4}\right)\\ -1,\frac{1}{{f}_{0}}\left(n+\frac{1}{4}\right)\le t < \frac{1}{{f}_{0}}\left(n+\frac{3}{4}\right)\end{array}\right.,n=0,\pm 1,\pm 2\ldots \ldots$$

It is evident from (1) that the inverter can also be considered a frequency mixer with a local oscillator ${g}(t)$.

By Fourier transform, ${g}(t)$ can be expressed as

3 $${g}(t) 	=\mathop{\sum}\limits_{n}\frac{4}{n\pi }\cos \left(2n\pi {f}_{0}t\right)=\mathop{\sum}\limits_{n}\frac{4}{n\pi }\cos \left(n{\omega }_{0}t\right),\\ n	 =1,3,5\ldots \ldots ,{\omega }_{0}=2\pi {f}_{0}$$

When the MCR is completely tuned to the resonant frequency ${f}_{0}$, the output current and output voltage remain in phase. Thus, the output current of the inverter can be expressed as

4 $${i}_{2}={I}_{2}\cos \left(2\pi {f}_{0}t\right)$$

where ${I}_{2}$ is the amplitude of the current.

The current ${i}_{2}$ induces a load side current, denoted as ${i}_{3}$ (Fig. 2b4),

5 $${i}_{3}={I}_{3}\cos \left(2\pi {f}_{0}t+\varphi \right)$$

where ${I}_{3}$ and $\varphi$ are the amplitude and phase of the current. In the MCR-WPT system with four coils, $\varphi$ is approximately $-\pi /2$ (see Methods).

On the load side, the current ${i}_{3}$ is rectified to dc, and the input voltage of the rectifier is forced to be a square wave (Fig. 2b5). This process is similar to the inverter process and can be expressed as

6 $${v}_{4}={v}_{3}\, \cdot \, g^{\prime} (t)$$

where ${v}_{3}$, ${v}_{4}$ are the input and output voltages of the rectifier, and $g^{\prime} (t)$ is a unit square wave function, defined as

7 $$g^{\prime} (t)=g(t+\varphi /{\omega }_{0})$$

Thus, the rectifier can also be considered a frequency mixer with a virtual local oscillator $g^{\prime} (t)$. Furthermore, $g^{\prime} (t)$ and $g(t)$ join a pair of synchronous oscillators with a phase shift *φ*, and this character is the foundation for the WPIDT system.

### Multiplexing inverter and rectifier as frequency mixers for integrated power and information transfer

A WPIDT system is a wireless system that transfers dc-integrated power and data from one side to another. Due to the synchronous nature of the inverter and rectifier, the information superimposed on the dc side of the front side can travel through the inverter, the MCR, and the rectifier to reach the dc side on the load side, and vice versa.

**Fig. 3:**
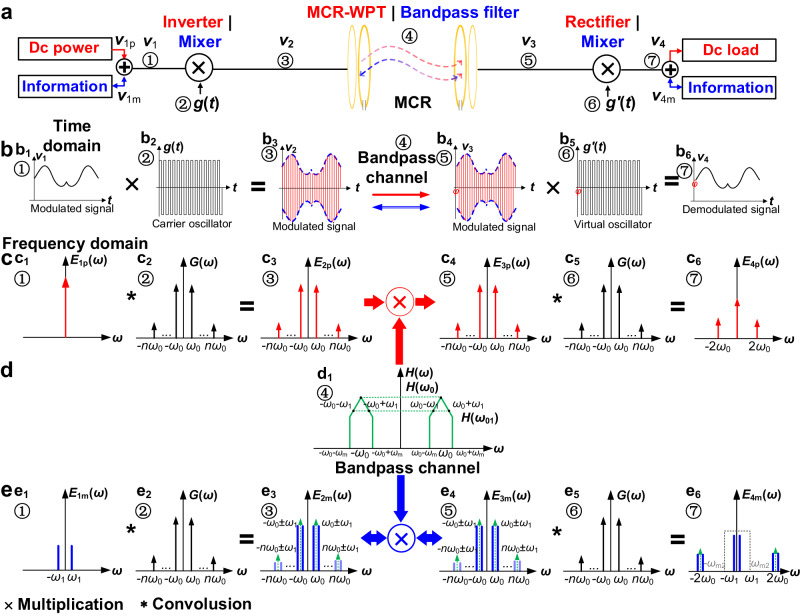
Waveforms and spectrum of power and information flow in WPIDT system via MCR. **a** Block diagram of the WPIDT system. *v*_1p_ denotes the dc voltage and *v*_1m_ denotes the information voltage, *v*_4m_ denotes the recovered information voltage. **b** Waveforms at position ①-⑦ and their relationship. b_1_ is the modulated voltage *v*_1_ at ①, b_2_ is the local oscillator *g*(*t*) at ②, b_3_ is the front side output voltage *v*_2_ at ③, red wave denotes the power and blue dash line denotes the information envelope, b_4_ is the load side modulated voltage *v*_3_ at ⑤, b_5_ is the virtual oscillator wave *g*^′^(*t*) at ⑥, and b_6_ is the demodulated signal *v*_4_ at ⑦. Red line denotes the power flow direction and blue line denotes the information transmission path. **c** Spectrum of power component at position ①-⑦ which marked in red. *E*_1p_(ω)~*E*_4p_(ω) refer to the power component, *G*(ω) refer to spectrum of the oscillator. *ω*_0_ denotes the switches frequency and *ω*_1_ denotes the data carrier frequency. **d** Transmission character of MCR channel at ④. *ω*_m_ denotes the pass band frequency. H(ω_0_) and H(ω_01_) denote the amplitude. **e** Spectrum of information at position ①-⑦ which marked in green. *E*_1m_(ω)~*E*_4m_(ω) refer to the information component which marked in blue. *ω*_m2_ denotes the pass band frequency of the demodulator.

Figure 3a depicts the function blocks of the WPIDT system, where the front-side inverter and load-side rectifier are simplified as a pair of frequency mixers. In downward communication, the information signal is transmitted at ${v}_{\rm1m}$ and received at ${v}_{\rm4m}$, whereas in upward communication, the information signal is transmitted at ${v}_{\rm 4m}$ and received at ${v}_{\rm1m}$. Notably, the communication channel is established based on the power channel and the amplitude of the information signal should be clearly less than the dc value.

The process of power and data transfer in the system can be divided into three stages: modulation, transmission, and demodulation. Consider the process with downward communication, i.e. transmitting data from the front side to the load side, as described in the following section.

The first stage is modulation, in which the power and information are modulated by the inverter. At position ① in Fig. 3a, the information modulated on the signal ${v}_{1m}$ is added to the dc source ${E}_{1}$. Assuming that ${v}_{\rm1m}$ is a two phase shift keying (2PSK) modulated signal, it can be described as ${{m}_{1}(t)=A}_{1}{d}_{1}(t)\sin (2\pi {f}_{1}t)$, where ${f}_{1}$ is the data carrier frequency, ${A}_{1}$ is the carrier amplitude, and ${d}_{1}(t)$ is a bipolar data sequence using −1 to represent 0. The input voltage of the inverter can be expressed as

8 $${v}_{1}={E}_{1}+{m}_{1}(t)$$

Considering the inverter’s switching frequency ${f}_{0}$ is much higher than the carrier frequency ${f}_{1}$, the signal ${v}_{1}$ is a narrowband signal with a bandwidth of ${B}_{{{{{{\rm{s}}}}}}}=2({f}_{1}+{B}_{{{{{{\rm{d}}}}}}})$, where ${B}_{{{{{{\rm{d}}}}}}}$ is the symbol rate of the data sequence.

As a mixer, the inverter multiplies the input voltage by a designed square wave with frequency ${f}_{0}$ at its output, and the relation between the input voltage ${v}_{1}$ and output voltage ${v}_{2}$ are illustrated in Fig. 3b3, and expressed as

9 $${v}_{2}=[{E}_{1}+{m}_{1}\left(t\right)]\cdot g\left(t\right)$$

where ${g}(t)$ is the unit square wave function defined in (2), and its spectrum is denoted as $G\left(\omega \right)$ shown in Fig. 3c2, c5, e2 and e5,

10 $$G\left(\omega \right)=\mathop{\sum}\limits_{n}\frac{2}{n\pi }\delta \left(\omega -n{\omega }_{0}\right),n=\pm 1,\pm 3,\pm 5\ldots \ldots .$$

Because a time domain multiplication equals a frequency domain convolution, the spectrum of ${v}_{2}$ can be expressed in frequency domain as

11 $${V}_{2}\left(\omega \right)={E}_{1}\cdot G\left(\omega \right)+\frac{1}{2}{A}_{1}[D\left(\omega -{\omega }_{0}\right)+D\left(\omega +{\omega }_{0}\right)]* G\left(\omega \right)$$

where $D(\omega )$ is the spectrum of the baseband signal ${d}_{1}(t)$. Ignoring the high harmonic of the square wave, it can be derived as

12 $${V}_{2}\left(\omega \right)=\, 	 {E}_{1{{{{{\rm{P}}}}}}}\left[\delta \left(\omega -{\omega }_{0}\right)+\delta \left(\omega +{\omega }_{0}\right)\right]\\ 	 +{M}_{1{{{{{\rm{M}}}}}}}\left[D\left(\omega +{\omega }_{0}+{\omega }_{1}\right)+D\left(\omega +{\omega }_{0}-{\omega }_{1}\right) \right. \\ 	 \, \, \left. +D\left(\omega -{\omega }_{0}+{\omega }_{1}\right)+D\left(\omega -{\omega }_{0}-{\omega }_{1}\right)\right].$$

where $${E}_{1{{{{{\rm{P}}}}}}}=\frac{2}{\pi }{E}_{1}$$ and $${M}_{1{{{{{\rm{M}}}}}}}=\frac{1}{\pi }{A}_{1}$$.

The spectrum ${V}_{2}\left(\omega \right)$ is shown in Fig. 3e3. It shows that the primary lobes of both power and data fall within a narrow frequency range. The passive MCR network, which consists of coils and compensation components, is a suitable bandpass channel for transmitting ${V}_{2}\left(\omega \right)$.

The second stage is transmission, in which the modulated power and information signals are transmitted through the MCR channel. To facilitate derivation, the transfer function of the bandpass channel $H(\omega )$ is assumed to be a linear-phase bandpass filter (Supplementary Method 1) that is symmetrical around ${\omega }_{0}$ in the ${V}_{2}$ spectrum, described as

13 $$\left\{\begin{array}{c}H(\omega )={{{{{\rm{|}}}}}}H\left(\omega \right){{{{{\rm{|}}}}}}{e}^{-j\left(\omega -{\omega }_{0}\right)\tau +j\varphi }\\ {{{{{\rm{|}}}}}}H\left(\omega +{\omega }_{0}\right){{{{{\rm{|}}}}}}={{{{{\rm{|}}}}}}H\left(\omega -{\omega }_{0}\right){{{{{\rm{|}}}}}}\end{array}\right.$$

Because the data bandwidth ${B}_{{{{{{\rm{d}}}}}}}$ is much small than ${f}_{0}$ and ${f}_{1}$, $H(\omega )$ can be assumed to be fixed within the frequency range of ${B}_{{{{{{\rm{d}}}}}}}$. Thus, the signal received on the load side at ⑤ can be derived as

14 $${V}_{3}\left(\omega \right)= \, 	 {V}_{2}\left(\omega \right)\times H\left(\omega \right)\approx {E}_{1{{{{{\rm{P}}}}}}}H\left({{{{{{\rm{\omega }}}}}}}_{0}\right)\left[\delta \left(\omega -{\omega }_{0}\right)+\delta \left(\omega +{\omega }_{0}\right)\right] \\ 	 +{M}_{1{{{{{\rm{M}}}}}}}[H(-{\omega }_{0}-{\omega }_{1})D\left(\omega +{\omega }_{0}+{\omega }_{1}\right)+H({-\omega }_{0}+{\omega }_{1})D\left(\omega +{\omega }_{0}-{\omega }_{1}\right) \\ 	 + H({\omega }_{0}-{\omega }_{1})D\left(\omega -{\omega }_{0}+{\omega }_{1}\right)+H({\omega }_{0}+{\omega }_{1})D\left(\omega -{\omega }_{0}-{\omega }_{1}\right)].$$

Figures 3c4 and e4 show, respectively, the power and information components of ${V}_{3}\left(\omega \right)$, where the bandpass filter removes the harmonics while retaining the fundamental components within the bandpass range.

The third stage is demodulation, in which the received power and information signals on the load side are multiplied with the virtual square wave oscillator ${g}^{\prime}(t)$. Similar to the inverter on the front side, the output signal ${V}_{4}$ is derived as

15 $${V}_{4}\left(\omega \right)=	 \frac{4}{{\pi }^{2}}{E}_{1{{{{{\rm{P}}}}}}}H\left({\omega }_{0}\right)\delta \left(\omega \right)\sin \varphi +\frac{4}{{\pi }^{2}}{M}_{1{{{{{\rm{M}}}}}}}\left|H\left({\omega }_{0}-{\omega }_{1}\right)\right|\\ 	 \cdot [D\left(\omega +{\omega }_{1}\right){e}^{j{\omega }_{1}\tau }+D\left(\omega -{\omega }_{1}\right){e}^{-j{\omega }_{1}\tau }]\sin \varphi .$$

where $\varphi$ is the phase shift between the rectifier on the load side and the inverter on the front side.

Equation (15) shows that ${V}_{4}(\omega )$ is comprised of a dc power component and an information component, and it is converted to the time domain as

16 $${v}_{4}={E}_{2}+{m}_{2}(t)$$

The output spectrums of the dc power component and the information component are shown in Fig. 3c6 and e6, respectively. The dc power is filtered and output to the load. The information component can be separated by a decouple network with resonant frequency ${f}_{1}$, and the recovered signal is shown in Fig. 3b6.

The analysis for upward communications (transmitting data from the load side to the front side) is identical to that for downward communication and is omitted. It should be pointed out here that the modulation method used in the system for downward communication can be described as the envelope modulation. However, this is not the case for the upward communication as the information is transmitted in the opposite direction of power transfer.

In summary, the power-electronics-based WPIDT system is comparable to a communication system in that it contains the three elements of a communication system: modulator (frequency mixer), communication channel (MCR) and demodulator (frequency mixer). The information signal with low frequency carrier, as well as the dc power, can pass through the MCR-WPT system, so the WPIDT system can be achieved by modulating the information on one dc side and receiving it on the other dc side. This method provides a perspective for power electronic converters and a research direction for integrated power and information transfer in WPT system.

### Communication channel characteristic of MCR-Based WPIDT system

The channel characteristics are essential for both power and information transmission. Given that the power transfer process has been extensively studied^7–11^, we focus on the features of the information transmission channel, including the signal gain and the channel bandwidth.

**Fig. 4:**
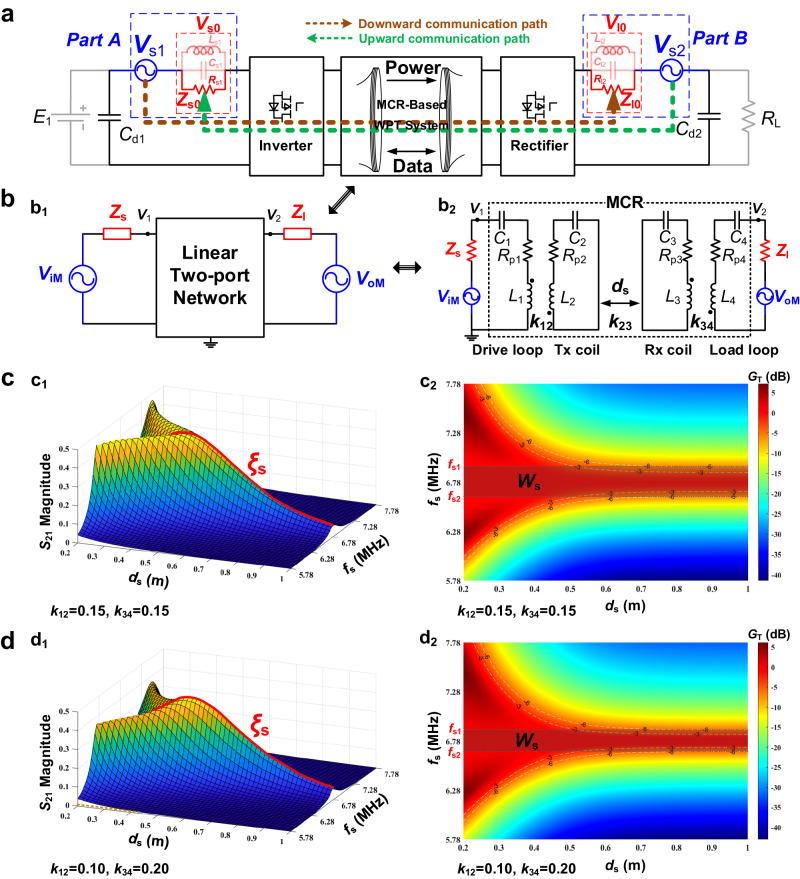
Channel characteristics of the MCR-based WPIDT system. **a** Simplified circuit of the MCR-based WPIDT system. *E*_1_ is the input dc source, *C*_d1_ and *C*_d2_ are the filter capacitance and *R*_L_ is the load resistance. *V*_s1_ and *V*_s2_ denote the data source, *V*_s0_ and *V*_l0_ denote the voltage of the data receiver. *Z*_s0_ and *Z*_l0_ denote the matching circuits, *R*_s1_ and *R*_l2_ are the resistance, *C*_s1_ and *C*_l2_ are the capacitance, *L*_s1_ and *L*_l2_ are the inductance. **b** Circuit model of the WPIDT system: b_1_ corresponds to a simplified linear two-port model, *V*_iM_ and *V*_oM_ denote the equivalent data source, *Z*_s_ and *Z*_l_ denote the equivalent impedance, *v*_1_ and *v*_2_ denote the signal output voltage. b_2_ corresponds to an equivalent model of MCR-based system. *C*_1_ ~ *C*_4_ denote the capacitance, *R*_p1_ ~ *R*_p4_ denote the resistance and *L*_1_ ~ *L*_4_ denote the inductance of resonators. *k*_12_, *k*_23_ and *k*_34_ denote the coupling coefficient of coils, *d*_s_ is the distance between *L*_2_ and *L*_3_. **c** Channel characteristic under symmetric coils: c_1_ reflects the transmission gain at different distances, and c_2_ shows the communication bandwidth of the system. *f*_s_ is the scanning frequency, ${\xi }_{{{{{{\rm{s}}}}}}}$ is the reference gain and *W*_s_ is the bandwidth. *S*_21_ is the scattering parameter. **d** Channel characteristic under asymmetric coils: d_1_ reflects the transmission gain at different distances, and d_2_ shows the communication bandwidth of the system.

To build a duplex communication model for the WPIDT system, two signal sources (${V}_{{{{{{\rm{s}}}}}}1}$ and ${V}_{{{{{{\rm{s}}}}}}2}$) and matching circuits (${Z}_{{{{{{\rm{s}}}}}}0}$ and ${Z}_{{{{{{\rm{l}}}}}}0}$) are introduced to the system, as depicted in Fig. 4a. In practical, ${Z}_{{{{{{\rm{s}}}}}}0}$ and ${Z}_{{{{{{\rm{l}}}}}}0}$ are parallel RLC circuits that resonate at frequency ${f}_{1}$ and have negligible effect on the power transfer at frequency ${f}_{0}$. The capacitors ${C}_{{{{{{\rm{d}}}}}}1}$ and ${C}_{{{{{{\rm{d}}}}}}2}$ on the front and load sides are large enough to be considered shorted at frequency ${f}_{1}$. In downward communication, ${V}_{{{{{{\rm{s}}}}}}1}$ is the signal source, ${V}_{{{{{{\rm{l}}}}}}0}$ is the received data signal, and ${Z}_{{{{{{\rm{s}}}}}}0}$ and ${Z}_{{{{{{\rm{l}}}}}}0}$ are the matching impedance on the transmitter side and receiver side respectively. In upward communication, the data signal is sent from ${V}_{{{{{{\rm{s}}}}}}2}$ and received at ${V}_{{{{{{\rm{s}}}}}}0}$.

Considering the frequency mixing effect of the inverter and the rectifier, the communication model of the system can be simplified as an equivalent circuit illustrated in Fig. 4b1 (see Supplementary Method 2 for proof), where ${V}_{{{{{{\rm{iM}}}}}}}$ is the modulated signal produced by mixing the signal source ${V}_{{{{{{\rm{s}}}}}}1}$ with the inverter’s switching function in downward communication process, ${V}_{{{{{{\rm{oM}}}}}}}$ is the modulated signal produced by mixing the signal source ${V}_{{{{{{\rm{s}}}}}}2}$ with the rectifier’s switching function in upward communication, and ${Z}_{{{{{{\rm{s}}}}}}}$ and ${Z}_{{{{{{\rm{l}}}}}}}$ are the equivalent impedance of ${Z}_{{{{{{\rm{s}}}}}}0}$ and ${Z}_{{{{{{\rm{l}}}}}}0}$, respectively. The relations between ${Z}_{{{{{{\rm{s}}}}}}}$, ${Z}_{{{{{{\rm{l}}}}}}}$ and ${Z}_{{{{{{\rm{s}}}}}}0}$, ${Z}_{{{{{{\rm{l}}}}}}0}$ through the inverter and rectifier converters are derived as follows (see Supplementary Method 2 for proof),

17 $$\left\{\begin{array}{c}{Z}_{{{{{{\rm{s}}}}}}}=\frac{8}{{\pi }^{2}}{Z}_{{{{{{\rm{s}}}}}}0}\\ {Z}_{{{{{{\rm{l}}}}}}}=\frac{8}{{\pi }^{2}}{Z}_{{{{{{\rm{l}}}}}}0}\end{array}\right.$$

In this work, ${Z}_{{{{{{\rm{s}}}}}}}$ and ${Z}_{{{{{{\rm{l}}}}}}}$ are carefully designed at 50 $\Omega$ (marked as ${R}_{0}$) to make the communication system consistent with the standard 50 $\Omega$ measurement environment^27^, which is helpful for vector network analyser (VNA) measurements.

The channel transmission characteristics of current MCR-based information and power simultaneous transfer system^19^ is highly correlated with the transfer distance ${d}_{{{{{{\rm{s}}}}}}}$. Thus, to analyze the information channel characteristics at different distances, the typical MCR circuit with four coils is presented in Fig. 4b2, which includes a drive coil ${L}_{1}$, a transmitter coil ${L}_{2}$, a receiver coil ${L}_{3}$, and a load coil ${L}_{4}$, with each coil connected by a resonant capacitor ${C}_{i}$, and a parasitic resistor ${R}_{{{{{{\rm{p}}}}}}i}$.

The coupling coefficients between ${L}_{1}$ and ${L}_{2}$, ${L}_{2}$ and ${L}_{3}$, and ${L}_{3}$ and ${L}_{4}$ are denoted by ${k}_{12}$, ${k}_{23}$, and ${k}_{34}$, respectively. For simplicity, the cross-coupling terms (${k}_{13}$, ${k}_{24}$, and ${k}_{14}$) are neglected in the following analysis. Typically,$\,{L}_{1}$ and ${L}_{2}$ are built into a single device, as are ${L}_{3}$ and ${L}_{4}$, so ${k}_{12}$ and ${k}_{34}$ are fixed. Thus, ${k}_{23}$ is the only coefficient that varies with the distances ${d}_{{{{{{\rm{s}}}}}}}$ between the transmitter and receiver.

Scattering parameters are an effective way to depict the properties of an MCR system and can be used to characterize the signal gain over the MCR channel^7,8^. The scattering parameters ${S}_{21}$ and ${S}_{12}$, which are twice the ratios of signal output voltages ${V}_{1}$ and ${V}_{2}$ to input signal voltages ${V}_{{{{{{\rm{iM}}}}}}}$ and ${V}_{{{{{{\rm{oM}}}}}}}$ in the presence of a 50 $\Omega$ matching resistor^8^ are obtained from Fig. 4b2 as follows (see Methods)

18 $$\left\{\begin{array}{c}{S}_{21}=\frac{{2V}_{2}}{{V}_{{{{{{\rm{iM}}}}}}}}=-\frac{2j{c}_{1}{\omega }^{3}{k}_{23}{R}_{{{{{{\rm{l}}}}}}}}{{c}_{2}{\omega }^{4}+{\omega }^{2}\left({c}_{3}+{c}_{4}{k}_{23}^{2}\right)+{c}_{5}}\\ {S}_{12}=\frac{{2V}_{1}}{{V}_{{{{{{\rm{oM}}}}}}}}=-\frac{2j{c}_{1}{\omega }^{3}{k}_{23}{R}_{{{{{{\rm{s}}}}}}}}{{c}_{2}{\omega }^{4}+{\omega }^{2}\left({c}_{3}+{c}_{4}{k}_{23}^{2}\right)+{c}_{5}}\end{array}\right.$$

where ${c}_{i}$ (*i* = 1, 2, 3, 4, 5) are constants when all parameters are fixed.

According to (18), ${{|S}}_{21}|={{|S}}_{12}|$, implying that the downward signal gain is equal to that of the upward signal gain, so we only consider ${S}_{21}$ in the following analysis.

${S}_{21}$ is a function with the variables frequency ${f}_{{{{{{\rm{s}}}}}}}$ and distance ${d}_{{{{{{\rm{s}}}}}}}$ that can be calculated mathematically. Since the coils on the load side can be symmetric or asymmetric with the coils on the front side, we calculate ${{|S}}_{21}|$ under the conditions of ${k}_{12}={k}_{34}$ and ${k}_{12} \, \ne \, {k}_{34}$, as shown in Fig. 4c1 and d1, respectively, using the parameters listed in Supplementary Table 1, Supplementary Note 1. These 3-D plots show ${{|S}}_{21}|$ as a function of frequency ${f}_{{{{{{\rm{s}}}}}}}$ and distance ${d}_{{{{{{\rm{s}}}}}}}$. According to Fig. 4c1 and d1, the frequency splitting occurs in over-coupled regime, which is similar to the power transfer characteristic, but in the under-coupled regime, ${{|S}}_{21}|$ reaches its maximum at the resonant frequency ${f}_{0}$ and is symmetrically distributed about ${f}_{0}$ in a fixed distance ${d}_{{{{{{\rm{s}}}}}}}$. Given that the medium-range MCR system operates in the under-coupled regime, ${{|S}}_{21}|$ at frequency ${f}_{0}$ is defined as the reference gain of the signal transmission in distance ${d}_{{{{{{\rm{s}}}}}}}$ and is denoted as ${\xi }_{{{{{{\rm{s}}}}}}}({d}_{{{{{{\rm{s}}}}}}})$, as shown in Fig. 4c1 and d1. To reflect the difference of the signal gain when frequency varies from the resonant frequency ${f}_{{{{{{\rm{s}}}}}}}$ at different distances ${d}_{{{{{{\rm{s}}}}}}}$, a normalized signal transmission gain ${G}_{{{{{{\rm{T}}}}}}}$ is defined as

19 $${G}_{{{{{{\rm{T}}}}}}}({f}_{{{{{{\rm{s}}}}}}},{d}_{{{{{{\rm{s}}}}}}}) \, \triangleq \, 20{\log }_{10}\frac{{{|S}}_{21}|}{{\xi }_{{{{{{\rm{s}}}}}}}({d}_{{{{{{\rm{s}}}}}}})}$$

As a bandpass channel, the MCR channel’s 6 dB bandwidth ${W}_{{{{{{\rm{c}}}}}}}({d}_{{{{{{\rm{s}}}}}}})$ in distance ${d}_{{{{{{\rm{s}}}}}}}$ is defined as the frequency range where ${G}_{{{{{{\rm{T}}}}}}}\ge -6{{{{{\rm{dB}}}}}}$, i.e. $\frac{{{|S}}_{21}|}{{\xi }_{{{{{{\rm{s}}}}}}}}\ge \frac{1}{2}$.

After normalization, the channel bandwidth can be illustrated by transforming Fig. 4c1 and d1 into Fig. 4c2 and d2, respectively. As shown in Fig. 4c2, the channel bandwidth decreases with increasing ${d}_{{{{{{\rm{s}}}}}}}$ at short range due to the frequency splitting, but stabilizes at medium range. Comparing Fig. 4c2 and Fig. 4d2, the channel bandwidth under asymmetric condition is slightly narrowed, but the variation trend of the gain remains unchanged. Therefore, to achieve communication over all available distances, the standard bandwidth ${W}_{{{{{{\rm{s}}}}}}}$ is defined as the narrowest bandwidth of the channel, which occurs at the maximum coils distance.

The upper limit cut-off frequency (equal to −6dB) is denoted by ${f}_{{{{{{\rm{s}}}}}}1}$, and the lower limit cut-off frequency (equal to −6dB) by ${f}_{{{{{{\rm{s}}}}}}2}$. Then, the standard bandwidth ${W}_{{{{{{\rm{s}}}}}}}$ can be written as

20 $${W}_{{{{{{\rm{s}}}}}}}={f}_{{{{{{\rm{s}}}}}}1}-{f}_{{{{{{\rm{s}}}}}}2}$$

Figure 4c2 illustrates the symmetric WPIDT system (${k}_{12}$ = ${k}_{34}\,$= 0.15), with ${f}_{{{{{{\rm{s}}}}}}1}$ is 6.93 MHz and ${f}_{{{{{{\rm{s}}}}}}2}$ is 6.63 MHz, indicating that ${W}_{{{{{{\rm{s}}}}}}}$ = 300 kHz. The channel characteristics of the WPIDT system in asymmetric condition (${k}_{12}=0.1$, ${k}_{34}$ = 0.2) is shown in Fig. 4d2. In comparison to Fig. 4c2, ${W}_{{{{{{\rm{s}}}}}}}$ is narrower (${W}_{{{{{{\rm{s}}}}}}}$ = 220 kHz).

${W}_{{{{{{\rm{s}}}}}}}$ is a crucial parameter for the performance of the MCR-based WPIDT system which limits the available spectrum of the data carrier, i.e., the spectrum of modulated signal ${v}_{1}(t)$ should fall inside the passband of the MCR channel, i.e. ${f}_{1}+{B}_{{{{{{\rm{d}}}}}}}\le \frac{{W}_{{{{{{\rm{s}}}}}}}}{2}$, where ${B}_{{{{{{\rm{d}}}}}}}$ is the signal bandwidth.

In summary, we analyse and simulate the communication characteristic of the MCR-WPT system and determine the bandwidth for communication, which is important for selecting the proper carrier frequency.

### Experimental part

**Fig. 5:**
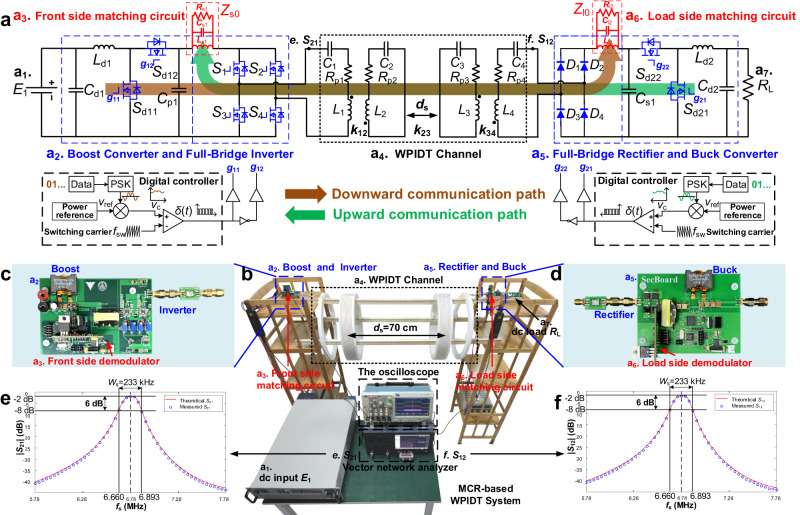
Prototype structure used in basic verification experiments. **a** Schematic of MCR-based WPIDT prototype, which includes a dc input ${E}_{1}$ (a_1_), a dc load ${R}_{{{{{{\rm{L}}}}}}}$ (a_7_), a boost/buck converter as a data generator (a_2_ and a_5_), two matching circuits (a_3_ and a_6_), a full-bridge inverter and a rectifier (a_2_ and a_5_), and MCR (a_4_). *L*_d1_ and *L*_d2_ denote inductance, *C*_d1_, *C*_d2_, *C*_p1_ and *C*_s1_ denote capacitance, *S*_d11_ ~ *S*_d22_ denote switches, *D*_1_ ~ *D*_4_ denote diodes. *Z*_s0_ and *Z*_l0_ denote the matching circuits, *R*_s1_ and *R*_l2_ are the resistance, *C*_s1_ and *C*_l2_ are the capacitance, *L*_s1_ and *L*_l2_ are the inductance. *L*_1_ ~ *L*_4_, *C*_1_ ~ *C*_4_ and *R*_p1_ ~ *R*_p4_ denote the inductance, capacitance and resistance of the resonators. *k*_12_, *k*_23_ and *k*_34_ denote the coupling coefficient between coils, *d*_s_ denotes the distance between *L*_2_ and *L*_3_. *V*_ref_ is the reference voltage, *V*_c_ is the signal vol*t*age and *δ*(*t*) is the PWM signal. **b** Photo of the prototype system corresponding to **a**. **c** Photo of the front-side circuit board. **d** Photo of the load-side circuit board. **e** Comparison between the theoretical and measured values of ${{|S}}_{21}|$ when ${d}_{{{{{{\rm{s}}}}}}}$ = 70 cm. *f*_s_ is the scanning frequency and *W*_s_ is the bandwidth. **f** Comparison of theoretical and measured values of ${{{{{\rm{|}}}}}}{S}_{12}|$ when ${d}_{{{{{{\rm{s}}}}}}}$ = 70 cm.

To verify the correctness of the scheme, a prototype system consisting a boost converter, a full-bridge inverter, MCR, a full-bridge rectifier, and a buck converter is constructed. The schematic diagram and the photo of the system are shown in Fig. 5a, b, respectively. The MCR consists of the drive coil, transmitter coil, receiver coil, and load coil, each of which has a 50 cm diameter and is aligned along an axis. The drive and load coils have 2 turns and ${L}_{1}={L}_{4}=2.18 \, \mu {{{{{\rm{H}}}}}}$, while the transmitter and receiver coils have 10 turns and ${L}_{2}={L}_{3}=90 \, \mu {{{{{\rm{H}}}}}}$. The transmitter and receiver coils are distributed compensated to increase the coils’ quality factor^28^. The distance between the drive coil and the transmitter coil is fixed at 15 cm, as is the distance between the receiver coil and the load coil. In the experiment, the distance ${d}_{{{{{{\rm{s}}}}}}}$ between the transmitter and receiver coils is fixed at 70 cm, so the transmission distance of the entire system is about 1 m.

In the prototype WPIDT system, the front-side boost converter functions as both a power regulator and a data transmitter for downward communication. As a power regulator, it regulates the output dc voltage based on load-side feedback; as a data transmitter, it transmits data signals modulated at frequency ${f}_{1}$. Similarly, the load-side buck converter serves as both a regulator for input impedance control and a data transmitter for upward communication. The power and signal dual modulation (PSDM) technique, which was presented in our previous works^20,21,23,24^ and is detailed in the method (see PSDM Method), is applied in the front-side boost and the load-side buck converters for simultaneous power conversion and data modulation. During communication, a micro-controller (STM32G431) transmits information by integrating the PSK modulated data signal into the control loop of the converters, thereby incorporating the data as a voltage perturbation into the power flow. GaN devices (GS61008T) are used to increase the switching frequency of the converters, and thus the bandwidth of the data signal. The switching frequency of the boost and buck is set to 800 kHz in this case, while the data carrier frequency is set to ${f}_{1}=100 \, {{{{{\rm{kHz}}}}}}$. In the second stage of power conversion, the full-bridge inverter converts dc to ac at ${f}_{0}=6.78$ MHz while modulating the low-frequency data carrier into the high-frequency power carrier band. Then, the ac power mixed with the data is transmitted to the load side. The boost works like an envelope tracker and the inverter functions as a productor on the front side during the downward communication, similar to the envelope modulation as mentioned before.

On the load side, a full-bridge rectifier restores the ac power to dc output. At the same time, the rectifier is also a frequency mixer. During downward communication, it shifts the frequency of data carrier from $6.78\pm 0.1$ MHz to 100 kHz. Then, the data signal is recovered through the matching circuit consisting of a parallel RLC network with a resonant frequency 100 kHz. Finally, after being filtered and amplified, the data signal is sampled and digitally demodulated by another micro-controller (STM32G431) on the load side. The second stage on the load side is the buck converter, which also employs PSDM strategy. During upward communication, it sends out data modulated with PSK signal, which are further modulated by the full-bridge rectifier to the high frequency of $6.78\pm 0.1$ MHz. After transmitting to the front side via the coils, the data signal is frequency-mixed with the inverter. The upward communication process on the front side is identical to the downward communication process on the load side.

In the PSDM method, the output power is controlled by the average duty cycle of the PWM signal, while the data signal is introduced by disturbing the duty cycle based on the modulated data signal. The maximum disturbance of duty cycle, as defined by perturbation depth $\eta$, is a parameter that indicates the strength of data carrier. The smaller the perturbation depth, the less impact the data signal has on power. To minimize the influence of the data signal on power transfer, we set $\eta =0.01$ in the experiments. The following experiments are conducted.

First, we evaluate the power transfer performance of the system. The target of the WPIDT system is to keep the output voltage on the load side at 48 V while maintaining optimal power transfer efficiency. The duty cycle of the buck converter is adjusted according to the load resistance, such that the input impedance of the buck is set at about 320 $\Omega$, the optimal value for power transfer. Meanwhile, the input voltage on the front side is fixed at 24 V and the boost converter is regulated to maintain a 48 V output voltage on the load side. The efficiency of power transfer when load resistances range from 50 $\Omega$ to 100 $\Omega$ (output power from 45 W to 23 W) is recorded in Supplementary Discussion 3, where the overall efficiency of the system is maintained above 62%. This experiment verifies that the WPIDT system efficiently transfer power.

Then, the parameters of the communication link are measured, and the communication performance is tested. Figure 5e, f shows the *S*-parameter sweep of the MCR channel by a VNA (E5061B), which corresponds to the theoretical calculation results and validates the channel model presented in this article. According to the scanning results of ${{|S}}_{21}|$ and ${{|S}}_{12}|$ (see Supplementary Discussion 2), when the signal power gain drops by 6 dB, the standard bandwidth ${W}_{{{{{{\rm{s}}}}}}}$ is 233 kHz. Therefore, the data carrier frequency is selected at 100 kHz (${f}_{1}=100 \, {{{{{\rm{kHz}}}}}}$), and the symbol rate is set to 20 kBaud (${B}_{{{{{{\rm{d}}}}}}}=20$ kBaud), which satisfies ${{f}_{1}+B}_{{{{{{\rm{d}}}}}}}\approx \frac{{W}_{{{{{{\rm{s}}}}}}}}{2}$.

**Fig. 6:**
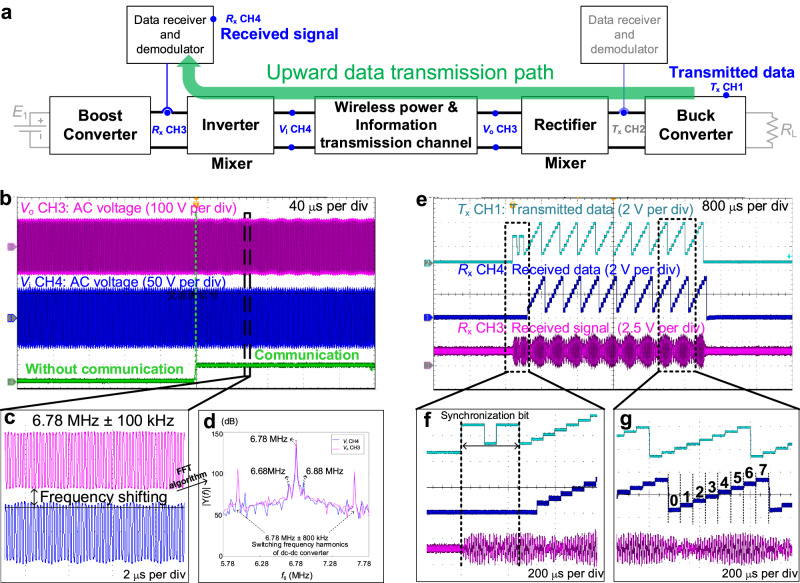
Waveform analysis of upward data transmission in MCR-based WPIDT system. **a** Block diagram of MCR-based WPIDT upward transmission path, the base band data is PSK-modulated and integrated into the dc power (*T*_x_ CH1) through the buck circuit, frequency-mixed by the rectifier and upconverted to the sideband of 6.78 MHz (*V*_o_ CH3), transmitted to the front side via the MCR channel (*V*_i_ CH4), frequency-mixed by the inverter and downconverted to the PSK-modulated signal (*R*_x_ CH3), and finally restored to original baseband data via filtering circuit and demodulation algorithms (*R*_x_ CH4). **b** The frequency-mixed signal before (*V*_o_ CH3) and after (*V*_i_ CH4) passing through the MCR, and the indication of communication mode. **c** Zoomed-in waveforms in communication mode. **d** Fast Fourier transform (FFT) | Y(*f*)| of the waveform in communication mode, with two points located 100 kHz away from 6.78 MHz, reflecting the frequency mixing effect of the rectifier. **e** Waveforms of transmitted and received data: transmitted baseband data (*T*_x_ CH1), received data (*R*_x_ CH4), and received signal before demodulation (*R*_x_ CH3). **f** Zoomed-in synchronization bits before a data frame. **g** Zoomed-in data frame coded by 8PSK with 60 kbps communication rate.

In upward communication, the block diagram of the MCR-based WPIDT system is shown in Fig. 6a. PSDM method is employed in the buck converter, with the switching frequency set to 800 kHz and the data carrier frequency set to 100 kHz. The data is modulated with 8DPSK and transmitted by the buck converter with $\eta =0.01$, and the corresponding communication waveforms are depicted in Fig. 6b–g. Figure 6b shows the waveforms with and without communication. As the amplitude of the data carrier is small, the impact of communication on power transfer is negligible. The communication waveform is zoomed in and presented in detail in Fig. 6c, and the spectrum of the modulated signal is shown in Fig. 6d, which is consistent with the analysis.

The demodulation result is compared with the transmitted data as shown in Fig. 6e. *R*_x_ CH3 is the waveform of the modulated signal that has reverted to the low-frequency carrier via the frequency mixing process. *R*_x_ CH4 represents the demodulated data output by the micro-controller’s digital-to-analog converter, and the detailed waveform is shown in Fig. 6f, g. *T*_x_ CH1 is the transmitted data on the load side, which is compared with the received data *R*_x_ CH4. It is noted that 8DPSK modulation is adopted in this experiment, so the communication rate is 60 kbps (within the maximum data rate of the WPIDT system, see Supplementary Discussion 4).

Finally, the impact of communication on power transfer is measured. Under the condition of perturbation depth $\eta =0.01$, the overall efficiency of the system during the communication fluctuates less than 1% (see Supplementary Discussion 3), demonstrating that the communication has a negligible effect on the power transfer efficiency.

Following the upward communication, we also conduct downward communication experiments. The specific experimental results including the Supplementary Figs. 7–9, which are provided in Supplementary Discussion 1, are consistent with the results of the theoretical analysis.

## Discussion

By multiplexing the inverter and rectifier as a pair of synchronous mixers, we propose the WPIDT system, in which the power and data are simultaneously modulated and transmitted. The principle of the WPIDT system is introduced, and a prototype system has been constructed to verify the scheme. Our work successfully demonstrates that power electronic converters, including ac/dc and dc/ac converters, have the capability of signal transmitting and modulating/demodulating. Information can be modulated onto the sideband of the power carrier and transmitted through the medium-range MCR.

This article implements the WPIDT system using a full bridge topology. However, it is important to note that the WPIDT idea may also be applied to other power-electronics-based WPT systems^10,29–31^, such as class-E circuits. Furthermore, this technology has the potential to be implemented in WPT systems with variable power frequencies, such as “parity-time symmetric” systems, because the carrier frequency has no effect on the result. Additionally, WPIDT technology is also applicable to conventionally low frequency IPT systems, but a power carrier with a higher frequency is preferable because it offers a wider communication bandwidth.

While this article introduces the WPIDT system, more study is necessary to explore important issues such as the Shannon channel capacity and the implementation for multiple receivers. We hope this article will inspire more investigation on these subjects.

## Methods

### KVL equations for WPIDT system

According to the WPT schematic diagram illustrated in Fig. 4b2, the matrix equation of the coupled system is expressed using Kirchhoff’s theorem as

21 $${{{{{\bf{U}}}}}}={{{{{\bf{Z}}}}}}{{{{{\bf{I}}}}}}$$

In downward communication, ${{{{{\bf{U}}}}}}={\left[{V}_{{{{{{\rm{iM}}}}}}}\:0\:0\:0\right]}^{T}$, while in upward communication, ${{{{{\bf{U}}}}}}={\left[0\:0\:0\:{V}_{{{{{{\rm{oM}}}}}}}\right]}^{T}$. The current ${{{{{\bf{I}}}}}}={\left[{I}_{1}\:{I}_{2}\:{I}_{3}\:{I}_{4}\right]}^{T}$, and the impedance equation is

22 $${{{{{\bf{Z}}}}}}{{{{{\boldsymbol{=}}}}}}\left[\begin{array}{cccc}{Z}_{1} & -j\omega {M}_{12}&0 & 0\\ -j\omega {M}_{12} & {Z}_{2}& -j\omega {M}_{23} & 0\\ 0 & -j\omega {M}_{23} & {Z}_{3} & -j\omega {M}_{34}\\ 0 & 0 &-j\omega {M}_{34}&{Z}_{4}\end{array}\right]$$

where,

23 $$\left\{\begin{array}{c}{Z}_{1}={R}_{{{{{{\rm{s}}}}}}}+{R}_{{{{{{\rm{p}}}}}}1}+{j\omega L}_{1}+\frac{1}{{j\omega C}_{1}}\\ {Z}_{2}={R}_{{{{{{\rm{p}}}}}}2}+{j\omega L}_{2}+\frac{1}{{j\omega C}_{2}}\hfill\\ {Z}_{3}={R}_{{{{{{\rm{p}}}}}}3}+{j\omega L}_{3}+\frac{1}{{j\omega C}_{3}}\hfill\\ {Z}_{4}={R}_{{{{{{\rm{l}}}}}}}+{R}_{{{{{{\rm{p}}}}}}4}+{j\omega L}_{4}+\frac{1}{{j\omega C}_{4}}\\ {M}_{{ij}}={k}_{{ij}}\sqrt{{L}_{i}{L}_{j}}\hfill\end{array}\right.$$

The gain transfer functions of the channel, which are the ratios of the receiver voltages ${V}_{1}$ and ${V}_{2}$ to the input signals ${V}_{{{{{{\rm{iM}}}}}}}$ and ${V}_{{{{{{\rm{oM}}}}}}}$, are determined by solving (21) and are denoted as

24 $$\left\{\begin{array}{c}\frac{{V}_{2}}{{V}_{{{{{{\rm{iM}}}}}}}}=-\frac{j{\omega }^{3}{k}_{12}{k}_{23}{k}_{34}{L}_{2}{{L}_{3}\sqrt{{L}_{1}{L}_{4}}R}_{{{{{{\rm{l}}}}}}}}{{k}_{12}^{2}{{k}_{34}^{2}{L}_{1}{L}_{2}{L}_{3}{L}_{4}\omega }^{4}\,+\,{\omega }^{2}\left({k}_{12}^{2}{L}_{1}{L}_{2}{Z}_{3}{Z}_{4}\,+\,{k}_{34}^{2}{L}_{3}{L}_{4}{Z}_{1}{Z}_{2}+{k}_{23}^{2}{L}_{2}{L}_{3}{Z}_{1}{Z}_{4}\right)\,+\,{Z}_{1}{Z}_{2}{Z}_{3}{Z}_{4}}\\ \frac{{V}_{1}}{{V}_{{{{{{\rm{oM}}}}}}}}=-\frac{j{\omega }^{3}{k}_{12}{k}_{23}{k}_{34}{L}_{2}{{L}_{3}\sqrt{{L}_{1}{L}_{4}}R}_{{{{{{\rm{s}}}}}}}}{{k}_{12}^{2}{{k}_{34}^{2}{L}_{1}{L}_{2}{L}_{3}{L}_{4}\omega }^{4}\,+\,{\omega }^{2}\left({k}_{12}^{2}{L}_{1}{L}_{2}{Z}_{3}{Z}_{4}\,+\,{k}_{34}^{2}{L}_{3}{L}_{4}{Z}_{1}{Z}_{2}\,+\,{k}_{23}^{2}{L}_{2}{L}_{3}{Z}_{1}{Z}_{4}\right)+{Z}_{1}{Z}_{2}{Z}_{3}{Z}_{4}}\end{array}\right.$$

Therefore, the constant parameters of (24) are combined as shown in (18).

When ${R}_{{{\rm{s}}}}$ equals to zero at frequency ${f}_{0}$, (21) can be represented as the KVL matrix equation of the MCR-based circuit shown in Fig. 2a. In this case, ${Z}_{i}(i={{{{\mathrm{1,2,3,4}}}}})$ is an ideal resistor, the denominator of (24) corresponds to a real value, while the numerator is an imaginary value. Hence, the phase shift $\varphi$ between the output and input voltages is $-\pi /2$.

### PSDM method

The dc-dc converter can be exploited as an information source, for dc power line communication or visible light communication^20–22^, making the system more compact and efficient. Power and signal dual modulation (PSDM) is a technology that modulates data and converts power in a converter simultaneously. PSDM can be implemented by two methods^24^. The first method, namely power and signal dual modulation with single carrier (PSDM-SC), employs the power carrier as the data carrier. The second method, namely power and signal dual modulation in control loop (PSDM-CL), superimposes a low-frequency signal on the power control loop as the data carrier.

**Fig. 7:**
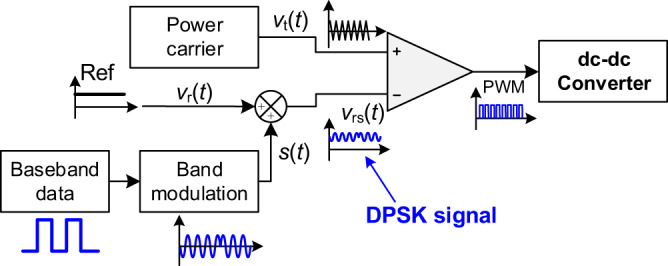
Data modulation processes in the converter using PSDM method. Blue line denotes the signal. *V*_t_(*t*) is the triangular carrier, *V*_r_(*t*) is the output of power control loop, s(*t*) is the data signal and *V*_rs_(*t*) is the power and data integrated signal.

In the prototype system, PSDM-CL method is used in both front side boost converter and load side buck converter, and is abbreviated as PSDM in the article. The data modulation processes in the boost/buck converter are shown in Fig. 7. First, the baseband data is modulated by a low frequency carrier as a perturbance signal $s(t)$. Conventional modulation methods, such as amplitude shift keying (ASK), frequency shift keying (FSK), phase shift keying (PSK), and multi-carrier modulation, can be employed in this process. In this experiment, 8DPSK modulation scheme is employed to increase the bitrate. Then, $s(t)$ is added to the output of power control loop ${v}_{{{{{{\rm{r}}}}}}}(t)$ as a power and data integrated signal ${v}_{{{{{{\rm{rs}}}}}}}(t)$. At last, ${v}_{{{{{{\rm{rs}}}}}}}(t)$ is compared with the triangular carrier ${v}_{{{{{{\rm{t}}}}}}}(t)$ to generate a PWM signal for the dc-dc converter. Using PSDM method, the data is merged into the PWM sequence. It should be noted that all the above processes are completed by software in a micro-controller.

The integrated data signal appears to be a disturbance of power. To indicate the strength of the disturbance, a parameter, perturbation depth $\eta$, is introduced to represent the maximum disturbance of duty cycle. Assuming the duty cycle of the power reference is *D*, the output duty cycle with perturbation depth $\eta$ and perturbation frequency (data carrier frequency) ${f}_{1}$ is expressed by

25 $${d}_{{{{{{\rm{g}}}}}}}=D+\eta \sin \left({2\pi f}_{1}t\right)$$

Typically, in a digital controlled dc-dc converter, the carrier frequency ${f}_{1}$ is selected as ${f}_{{{{{{\rm{s}}}}}}}/N$ (*N* is an integer), where ${f}_{{{{{{\rm{s}}}}}}}$ is the switching frequency of the converter.

Thus, the data carrier can be represented by a sequence of duty cycle. For example, when $D=0.5$, $\eta =0.01$, and $N=8$, the duty cycle sequence in a period of data carrier is {0.5, 0.507, 0.51, 0.507, 0.5, 0.493, 0.49, 0.493}. Furthermore, 8PSK modulation can be achieved by selecting the following duty cycle sequence.

Phase 0: {0.5, 0.507, 0.51, 0.507, 0.5, 0.493, 0.49, 0.493},

Phase $$\frac{\pi }{4}$$: {0.507, 0.51, 0.507, 0.5, 0.493, 0.49, 0.493, 0.5},

Phase $$\frac{\pi }{2}$$: {0.51, 0.507, 0.5, 0.493, 0.49, 0.493, 0.5, 0.507},

Phase $$\frac{3\pi }{4}$$: {0.507, 0.5, 0.493, 0.49, 0.493, 0.5, 0.507, 0.51},

Phase $$\pi$$: {0.5, 0.493, 0.49, 0.493, 0.5, 0.507, 0.51, 0.507},

Phase $$\frac{5\pi }{4}$$: {0.493, 0.49, 0.493, 0.5, 0.507, 0.51, 0.507, 0.5},

Phase $$\frac{3\pi }{2}$$: {0.49, 0.493, 0.5, 0.507, 0.51, 0.507, 0.5, 0.493},

Phase $$\frac{7\pi }{4}$$: {0.493, 0.5, 0.507, 0.51, 0.507, 0.5, 0.493, 0.49}.

In conclusion, PSDM in the WPIDT system combines power and data without requiring an additional circuit.

### Synchronization and demodulation method

In a communication system employing m-ary PSK modulation, the carrier and frame synchronization between the transmitter and receiver is of the critical importance^12^. In the experiment system, we incorporate the synchronization method into demodulation processing. The implementation of the synchronization and demodulation method is discussed as follows,

Initially, the MCU on the receiver continuously samples the input signal in a predetermined interval, followed by the windowed discrete Fourier transform (DFT) processing^20^. The general DFT algorithm is expressed as follows,

26 $$X(k)=\mathop{\sum }\limits_{n=0}^{{NM}-1}x(n){e}^{-j\frac{2n\pi }{M}}$$

where *N* is the carrier frequency cycle number in the sample window, *M* is the sampled number per carrier cycle, and {*x*(0), *x*(1), …, *x*(*NM* − 1)} is the sampled data sequence. It should be noted that the data sequence *x*(*n*) is updated with a sliding window of the DFT^20^. Consequently, the amplitude and phase of the signal are obtained in real time. Assuming that the frequency offset between the transmitter and receiver can be ignored, the phase difference between the transmitter and receiver is fixed over the duration of a frame.

**Fig. 8:**
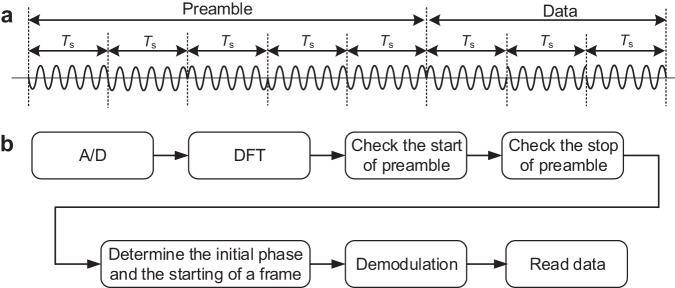
The implementation of the synchronization and demodulation method. **a** Frame structure. The dash line refers to the spacing of *T*_s_. **b** Synchronization and demodulation procedure.

To synchronize the transmitter and receiver, a preamble of five synchronization symbols is used, as shown in Fig. 8a. The phases of the synchronization symbols are set to {π, π, 0, π, π}, thus the receiver can detect a phase shift of 180 degrees twice during the preamble interval. On the basis of the moment of phase shifting, the initial phase of the received signal and the beginning of a frame are determined and subsequently employed for demodulation. The corresponding procedure is illustrated in Fig. 8b.

### Supplementary information

Supplementary Information

## Data Availability

The source data underlying Fig. 5e, f are available at Figshare: https://figshare.com/articles/dataset/Source_Data_zip/24587451. The additional data that support the findings of this study are available from the corresponding author upon reasonable request.
