# Supplementary material for: Wireless power and information dual transfer system via magnetically coupled resonators
Source: Commun Eng. 2024 Jan 8;3:8. doi: 10.1038/s44172-023-00154-4 (PMC10955837; doi:10.1038/s44172-023-00154-4)
Supplement: Supplementary file 1 — Supplementary Information [file 44172_2023_154_MOESM1_ESM.pdf]

# Supplementary Methods

## Supplementary Method 1: Linear Phase System

Consider a system with no phase distortion according to Supplementary reference 1, i.e. the filter's phase shift is linear. Supplementary Fig. 1 depicts a typical transfer function. The linear phase system has the following analytical form,

$$H(\omega) = |H(\omega)|\, e^{-j(\omega\tau-\varphi)}\,. \tag{a1}$$

The impulse response of this system is written as,

$$h(t) = \frac{1}{2\pi}\int_{-\infty}^{\infty}|H(\omega)|e^{-j(\omega\tau-\varphi)}e^{j\omega t}\,d\omega = \frac{1}{\pi}\int_{0}^{\infty}|H(\omega)|sin[\omega(t-\tau+\varphi/\omega)]\,d\omega\,. \tag{a2}$$

This impulse response $h(t)$ is symmetric about τ, written as,

$$h(t+\tau) = h(t-\tau)\,. \tag{a3}$$

At $t=\tau$, $h(t)$ reaches its maximum value, that is

$$h(\tau) = \frac{1}{\pi}\int_{0}^{\infty}|H(\omega)|\,sin\varphi d\omega\,. \tag{a4}$$

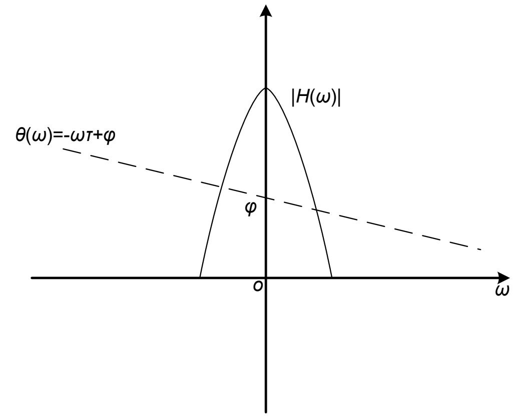


**Supplementary Fig. 1 A typical transfer function of a linear phase system.** *θ*(*ω*) denotes rotation phase shift of the filter and |*H*(*ω*)| denotes the amplitude shift of the filter.

When considering the bandpass filtering characteristics under the condition of 6.78MHz, $\omega \pm \omega_0$ can be used to replace $\omega$.

**Supplementary reference 1:** Gabel, R. A., & Roberts, R. A., *Signals and linear systems.* (John Wiley & Sons, Inc., 1973).

## Supplementary Method 2: Signal Transmission Characteristic of the WPIDT System with Full-bridge Inverter

In the WPIDT system, the full bridge inverter is also a frequency mixer. To investigate the signal transmission characteristic of the mixer, the WPIDT circuit is simplified as an equivalent RLC series circuit, where $L_{\mathrm{e}}$, $C_{\mathrm{e}}$ and $R_{\mathrm{eq}}$ represent the equivalent inductance, capacitance, and resistance, respectively. The equivalent circuit is assumed to meet the following requirements,

$$\omega_0^2 = \frac{1}{L_{\mathrm{e}}C_{\mathrm{e}}}\,. \tag{b1}$$

where $\omega_0$ is the switching frequency of the inverter, i.e. the local frequency of the mixer.

The equivalent circuit of the WPIDT with a full-bridge converter is shown in Supplementary Fig. 2, which is divided into low frequency (LF) and high frequency (HF) parts. The LF part contains a sinusoidal source $v_{\mathrm{s}}$ with frequency $\omega_1$ and a resistor $Z_{\mathrm{s0}}$. The inverter operated as a frequency mixer, multiplying the signal with frequency $\omega_1$ by a unit square wave function with frequency $\omega_0$ ($\omega_0 \gg \omega_1$) and upconverting the signal frequency to $\omega_0 \pm \omega_1$. On the HF side, the output of the mixer $v_{\mathrm{e}}$ is a HF signal with two different frequency components, denoted by $v_{\mathrm{e+}}$ and $v_{\mathrm{e-}}$. The derivation is as follows.

The inputs of the mixer are

$$v_{\mathrm{g}} = V_{\mathrm{g}}sin(\omega_1 t), \tag{b2}$$

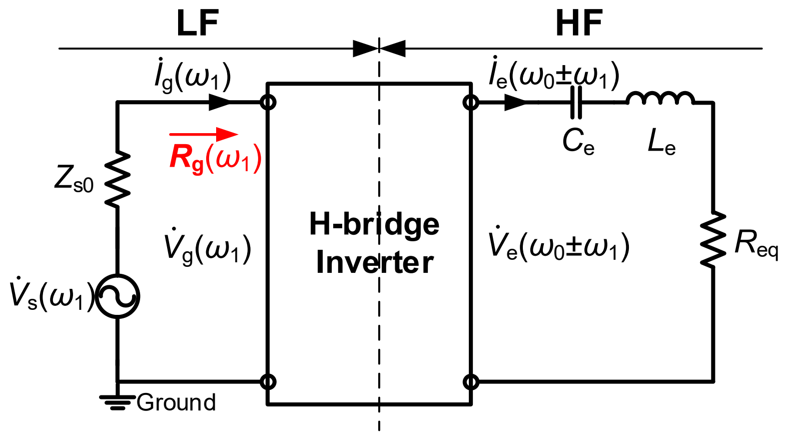


**Supplementary Fig. 2 Simplified equivalent circuit of the WPIDT system.** $\dot{V}_s(\omega_1)$ denotes signal voltage, $Z_{s0}$ denotes matching resistance, $\dot{I}_g(\omega_1)$ denotes low frequency (LF) side current, $R_g(\omega_1)$ denotes LF side equivalent resistance, $\dot{V}_g(\omega_1)$ denotes input voltage of mixers, $\dot{V}_e(\omega_1 \pm \omega_0)$ denotes high frequency (HF) side voltage, $\dot{I}_e(\omega_1 \pm \omega_0)$ denotes HF side current, $L_e$ denotes inductance, $C_e$ denotes capacitance and $R_{eq}$ denotes resistance.

$$g(t) = \begin{cases} 1, & \frac{1}{f_0}\left(n - \frac{1}{4}\right) \le t < \frac{1}{f_0}\left(n + \frac{1}{4}\right) \\ -1, & \frac{1}{f_0}\left(n + \frac{1}{4}\right) \le t < \frac{1}{f_0}\left(n + \frac{3}{4}\right) \end{cases}. \tag{b3}$$

Ignoring the high harmonic of the square wave, the output of the mixer is

$$v_e = v_g \cdot g(t) = \frac{2}{\pi} V_g[\sin(\omega_0 + \omega_1)t + \sin(\omega_0 - \omega_1)t] = v_{e+} + v_{e-}. \tag{b4}$$

In phasor, $v_g$, $v_{e+}$ and $v_{e-}$ are represented as $\dot{V}_g(\omega_1)$, $\dot{V}_{e+}(\omega_1 + \omega_0)$ and $\dot{V}_{e-}(\omega_1 - \omega_0)$, respectively.

The HF current $i_{e+}$ and $i_{e-}$ introduced by $\dot{V}_{e+}(\omega_1 + \omega_0)$ and $\dot{V}_{e-}(\omega_1 - \omega_0)$ can be calculated as

$$\begin{cases} \dot{I}_{e+}(\omega_0 + \omega_1) = \dfrac{\frac{2}{\pi} V_g}{j(\omega_0 + \omega_1) L_e \left[1 - \frac{\omega_0^2}{(\omega_0 + \omega_1)^2}\right] + R_{eq}} \\ \dot{I}_{e-}(\omega_0 - \omega_1) = \dfrac{\frac{2}{\pi} V_g}{j(\omega_0 - \omega_1) L_e \left[1 - \frac{\omega_0^2}{(\omega_0 - \omega_1)^2}\right] + R_{eq}} \end{cases}. \tag{b5}$$

Since the switching function $g(t)$ is composed of 1 and -1, multiplying or dividing by $g(t)$ has the same result.

Therefore, on the LF side, the current $i_g$ is obtained as

$$i_g = \frac{i_{e+} + i_{e-}}{g(t)} = [i_{e+} + i_{e-}] \cdot g(t) \approx [i_{e+} + i_{e-}] \cdot \frac{4}{\pi} \cos(\omega_0 t). \tag{b6}$$

The frequency $\omega_1$ component of $i_g$ is represented in phasor as $\dot{I}_g(\omega_1)$ and derived as

$$\dot{I}_g(\omega_1) = \frac{4}{\pi^2}\left[\frac{V_g}{j(\omega_0 + \omega_1) L_e \left[1 - \frac{\omega_0^2}{(\omega_0 + \omega_1)^2}\right] + R_{eq}} + \frac{V_g}{j(\omega_0 - \omega_1) L_e \left[1 - \frac{\omega_0^2}{(\omega_0 - \omega_1)^2}\right] + R_{eq}}\right] = \dot{I}_{g+}(\omega_1) + \dot{I}_{g-}(\omega_1). \tag{b7}$$

The current $\dot{I}_g(\omega_1)$ is divided into two parts, represented by $\dot{I}_{g+}(\omega_1)$ and $\dot{I}_{g-}(\omega_1)$ respectively, as shown in Supplementary Fig. 3.

The current $\dot{I}_{g+}(\omega_1)$ and $\dot{I}_{g-}(\omega_1)$ are orthogonally decomposed, with the real and imaginary parts deduced as

$$\dot{I}_{g+}(\omega_1): \begin{cases} Re[\dot{I}_{g+}(\omega_1)] = \dfrac{4V_g}{\pi^2} \dfrac{R_{eq}}{\left\{(\omega_0 + \omega_1) L_e \left[1 - \frac{\omega_0^2}{(\omega_0 + \omega_1)^2}\right]\right\}^2 + R_{eq}^2} \\ Im[\dot{I}_{g+}(\omega_1)] = \dfrac{-4V_g}{\pi^2} \dfrac{j(\omega_0 + \omega_1) L_e \left[1 - \frac{\omega_0^2}{(\omega_0 + \omega_1)^2}\right]}{\left\{(\omega_0 + \omega_1) L_e \left[1 - \frac{\omega_0^2}{(\omega_0 + \omega_1)^2}\right]\right\}^2 + R_{eq}^2} \end{cases}, \tag{b8}$$

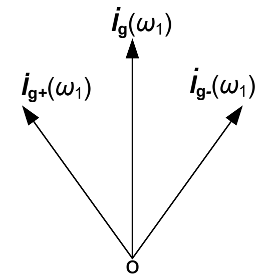


**Supplementary Fig. 3 The phasor of the current on the LF side.** $\dot{I}_{\mathrm{g}}(\omega_1)$, $\dot{I}_{\mathrm{g+}}(\omega_1)$ and $\dot{I}_{\mathrm{g-}}(\omega_1)$ denotes low frequency (LF) side currents.

$$\dot{I}_{\mathrm{g-}}(\omega_1):\begin{cases} Re[\dot{I}_{\mathrm{g-}}(\omega_1)] = \frac{4V_{\mathrm{g}}}{\pi^2}\frac{R_{\mathrm{eq}}}{\left\{(\omega_0-\omega_1)L_{\mathrm{e}}\left[1-\frac{\omega_0^2}{(\omega_0-\omega_1)^2}\right]\right\}^2+R_{\mathrm{eq}}^2} \\ Im[\dot{I}_{\mathrm{g-}}(\omega_1)] = \frac{-4V_{\mathrm{g}}}{\pi^2}\frac{j(\omega_0-\omega_1)L_{\mathrm{e}}\left[1-\frac{\omega_0^2}{(\omega_0-\omega_1)^2}\right]}{\left\{(\omega_0-\omega_1)L_{\mathrm{e}}\left[1-\frac{\omega_0^2}{(\omega_0-\omega_1)^2}\right]\right\}^2+R_{\mathrm{eq}}^2} \end{cases}. \tag{b9}$$

Combine the real and imaginary parts of $\dot{I}_{\mathrm{g+}}(\omega_1)$ and $\dot{I}_{\mathrm{g-}}(\omega_1)$, respectively, to obtain

$$\begin{aligned} Re[\dot{I}_{\mathrm{g+}}(\omega_1)] + Re[\dot{I}_{\mathrm{g-}}(\omega_1)] &= \frac{4V_{\mathrm{g}}R_{\mathrm{eq}}}{\pi^2}\left[\frac{1}{\left\{(\omega_0+\omega_1)L_{\mathrm{e}}\left[1-\frac{\omega_0^2}{(\omega_0+\omega_1)^2}\right]\right\}^2+R_{\mathrm{eq}}^2}+\frac{1}{\left\{(\omega_0-\omega_1)L_{\mathrm{e}}\left[1-\frac{\omega_0^2}{(\omega_0-\omega_1)^2}\right]\right\}^2+R_{\mathrm{eq}}^2}\right] \\ &= \frac{4V_{\mathrm{g}}R_{\mathrm{eq}}}{\pi^2}\left[\frac{2R_{\mathrm{eq}}^2+\left\{\left[\frac{\omega_1^2+2\omega_0\omega_1}{\omega_0+\omega_1}\right]^2+\left[\frac{\omega_1^2-2\omega_0\omega_1}{\omega_0-\omega_1}\right]^2\right\}L_{\mathrm{e}}^2}{\left\{\left[\frac{\omega_1^2+2\omega_0\omega_1}{\omega_0+\omega_1}L_{\mathrm{e}}\right]^2+R_{\mathrm{eq}}^2\right\}\left\{\left[\frac{\omega_1^2-2\omega_0\omega_1}{\omega_0-\omega_1}L_{\mathrm{e}}\right]^2+R_{\mathrm{eq}}^2\right\}}\right], \end{aligned} \tag{b10}$$

$$\begin{aligned} Im[\dot{I}_{\mathrm{g+}}(\omega_1)] + Im[\dot{I}_{\mathrm{g-}}(\omega_1)] &= \frac{-4jV_{\mathrm{g}}L_{\mathrm{e}}}{\pi^2}\left[\frac{(\omega_0+\omega_1)\left[1-\frac{\omega_0^2}{(\omega_0+\omega_1)^2}\right]}{\left\{(\omega_0+\omega_1)L_{\mathrm{e}}\left[1-\frac{\omega_0^2}{(\omega_0+\omega_1)^2}\right]\right\}^2+R_{\mathrm{eq}}^2}+\frac{(\omega_0-\omega_1)\left[1-\frac{\omega_0^2}{(\omega_0-\omega_1)^2}\right]}{\left\{(\omega_0-\omega_1)L_{\mathrm{e}}\left[1-\frac{\omega_0^2}{(\omega_0-\omega_1)^2}\right]\right\}^2+R_{\mathrm{eq}}^2}\right] \\ &= \frac{-4jV_{\mathrm{g}}L_{\mathrm{e}}}{\pi^2}\left[\frac{\left(2\omega_0-\frac{\omega_0^2}{\omega_0+\omega_1}-\frac{\omega_0^2}{\omega_0-\omega_1}\right)\left\{\left[\frac{\omega_1^2+2\omega_0\omega_1}{\omega_0+\omega_1}L_{\mathrm{e}}\right]^2+R_{\mathrm{eq}}^2\right\}\left\{\left[\frac{\omega_1^2-2\omega_0\omega_1}{\omega_0-\omega_1}L_{\mathrm{e}}\right]^2+R_{\mathrm{eq}}^2\right\}}{\left\{\left[\frac{\omega_1^2+2\omega_0\omega_1}{\omega_0+\omega_1}L_{\mathrm{e}}\right]^2+R_{\mathrm{eq}}^2\right\}\left\{\left[\frac{\omega_1^2-2\omega_0\omega_1}{\omega_0-\omega_1}L_{\mathrm{e}}\right]^2+R_{\mathrm{eq}}^2\right\}}\right]. \end{aligned} \tag{b11}$$

When $\omega_0 \gg \omega_1$, the real part and the imaginary are simplified to

$$Re[\dot{I}_{\mathrm{g+}}(\omega_1)] + Re[\dot{I}_{\mathrm{g-}}(\omega_1)] = \frac{8V_{\mathrm{g}}}{\pi^2}\frac{R_{\mathrm{eq}}}{(2\omega_1L_{\mathrm{e}})^2+R_{\mathrm{eq}}^2}, \tag{b12}$$

$$Im[\dot{I}_{\mathrm{g+}}(\omega_1)] + Im[\dot{I}_{\mathrm{g-}}(\omega_1)] \approx 0. \tag{b13}$$

Then, the current on the LF side contains only the real part component, and the LF equivalent resistance $R_{\mathrm{g}}(\omega_1)$ is

$$R_{\mathrm{g}}(\omega_1) = \frac{\dot{V}_{\mathrm{g}}(\omega_1)}{\dot{I}_{\mathrm{g}}(\omega_1)} = \frac{V_{\mathrm{g}}}{Re[\dot{I}_{\mathrm{g+}}(\omega_1)]+Re[\dot{I}_{\mathrm{g-}}(\omega_1)]} = \frac{\pi^2}{8}\left(\frac{4\omega_1^2L_{\mathrm{e}}^2}{R_{\mathrm{eq}}}+R_{\mathrm{eq}}\right). \tag{b14}$$

Supplementary Fig. 4a shows the equivalent circuit on the LF side, which can also be converted to the HF side. According to (b4), the output of the mixer is

$$\begin{aligned} \dot{V}_{\mathrm{e}} &= \dot{V}_{\mathrm{e+}}(\omega_1+\omega_0) + \dot{V}_{\mathrm{e-}}(\omega_1-\omega_0) \\ &= \frac{2}{\pi}[\dot{V}_{\mathrm{g}}(\omega_0+\omega_1) + \dot{V}_{\mathrm{g}}(\omega_0-\omega_1)] = \frac{\frac{4\omega_1^2L_{\mathrm{e}}^2}{R_{\mathrm{eq}}}+R_{\mathrm{eq}}}{\frac{8}{\pi^2}Z_{\mathrm{s0}}+\left(\frac{4\omega_1^2L_{\mathrm{e}}^2}{R_{\mathrm{eq}}}+R_{\mathrm{eq}}\right)}\cdot\frac{2}{\pi}[\dot{V}_{\mathrm{s}}(\omega_0+\omega_1)+\dot{V}_{\mathrm{s}}(\omega_0-\omega_1)]. \end{aligned} \tag{b15}$$

From (b15), the equivalent HF output voltage of the mixer is $\frac{2}{\pi}[\dot{V}_{\mathrm{s}}(\omega_0+\omega_1)+\dot{V}_{\mathrm{s}}(\omega_0-\omega_1)]$, and the equivalent impedance $Z_{\mathrm{s}}$ is equal to $\frac{8}{\pi^2}Z_{\mathrm{s0}}$, as shown in Supplementary Fig. 4b.

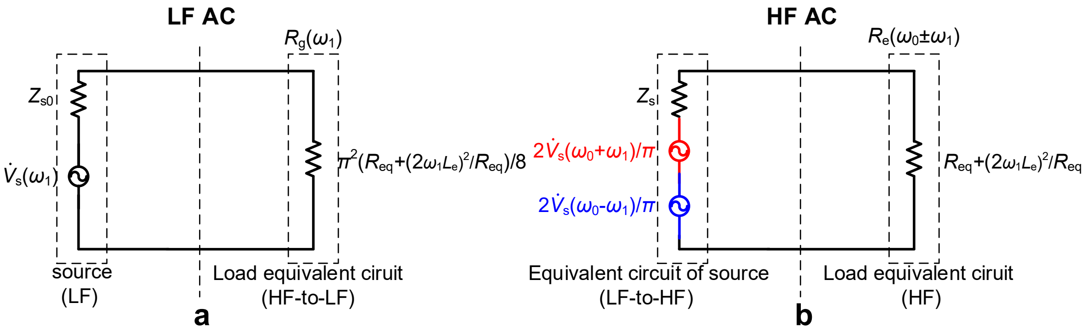


**Supplementary Fig. 4 Equivalent two-port circuit. a,** Low-frequency AC (LF AC) model. $\dot{V}_s(\omega_1)$ denotes signal voltage, $Z_{s0}$ denotes matching resistance, $R_g(\omega_1)$ denotes LF side equivalent resistance, $L_e$ denotes inductance and $R_{eq}$ denotes resistance; **b,** High-frequency AC (HF AC) model. $\dot{V}_s(\omega_1 \pm \omega_0)$ denotes high frequency (HF) side equivalent voltage of source, $Z_s$ denotes equivalent matching resistance, $R_e(\omega_1 \pm \omega_0)$ denotes HF resistance.

# Supplementary Notes

## Supplementary Note 1: Experimental parameters

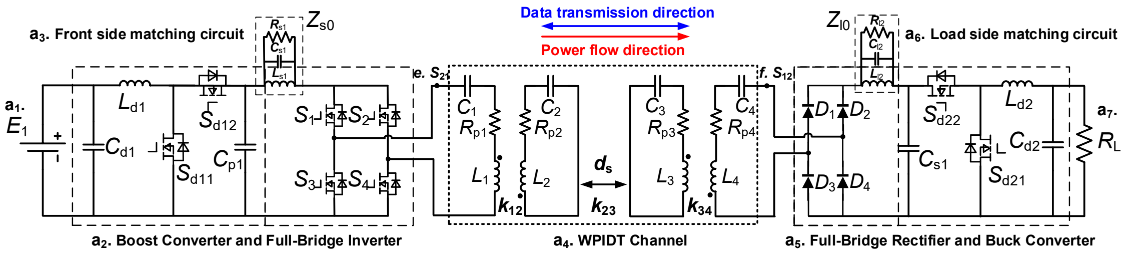


**Supplementary Fig. 5 Prototype structure used in experiments.** $E_1$ denotes input dc source, $R_L$ denotes load resistance, $L_{d1}$ and $L_{d2}$ denote inductance, $C_{d1}$, $C_{d2}$, $C_{p1}$ and $C_{s1}$ denote capacitance, $S_{d11}$~$S_{d22}$ denote switches, $D_1$~$D_4$ denote diodes. $Z_{s0}$ and $Z_{l0}$ denote the matching circuit, $R_{s1}$ and $R_{l2}$ are the resistance, $C_{s1}$ and $C_{l2}$ are the capacitance, $L_{s1}$ and $L_{l2}$ are the inductance. $L_1$~$L_4$, $C_1$~$C_4$ and $R_{p1}$~$R_{p4}$ denote the inductance, capacitance and resistance of the resonators. $k_{12}$, $k_{23}$ and $k_{34}$ denote the coupling coefficient between coils, $d_s$ denotes the distance between $L_2$ and $L_3$.

The system parameters in Supplementary Fig. 5 are shown in Supplementary Table 1.

Supplementary Table 1 Parameters of the MCR-based WPIDT system

| Converter | Symbol | Parameter | Value/Type |
|---|---|---|---|
| Front-side boost converter | $f_{sw}$ | Switch frequency | 800kHz |
| | $S_{d11}, S_{d12}$ | Switches | GS61008T |
| | $L_{d1}$ | Inductance | 10uH |
| | $C_{d1}$ | Capacitance | 200uF |
| | $C_{p1}$ | Capacitance | 200nF |
| MCR-based WPT system | $S_1$~$S_4$ | Switches | EPC2007C |
| | $D_1$~$D_4$ | Diodes | STPSC406B |
| | $L_1/L_4$ | Drive/load inductance | 2.18uH |
| | $L_2/L_3$ | Tx/Rx inductance | 90uH |
| | $f_0$ | Resonant frequency | 6.78MHz |
| | $R_{p1}/R_{p4}$ | Drive/load Winding resistance | 265mΩ/260mΩ |
| | $R_{p2}/R_{p3}$ | Drive/load Winding resistance | 5.89Ω/5.88Ω |
| | $d_{12}/d_{34}$ | Distance between $L_1$ and $L_2$/($L_3$ and $L_4$) | 150mm |
| | $d_s$ | Distance between $L_2$ and $L_3$ | 700mm |
| Load-side buck converter | $f_{sw}$ | Switch frequency | 800kHz |
| | $S_{d21}, S_{d22}$ | Switches | GS61008T |
| | $L_{d2}$ | Inductance | 10uH |
| | $C_{d2}$ | Capacitance | 200uF |
| | $C_{s1}$ | Capacitance | 200nF |
| | $R_L$ | Load | 50~100Ω |
| Matching circuits | $L_{s1}/L_{l2}$ | Inductance | 1uH/1uH |
| | $C_{s1}/C_{l2}$ | Capacitance | 2.54uF/2.54uF |
| | $R_{s1}/R_{l2}$ | Matching resistance | 61.2Ω |

# Supplementary Discussions

## Supplementary Discussion 1: Data transmission waveforms

The waveforms of the upward communication process tested at 100kHz (data carrier frequency) are provided in Supplementary Fig. 6 of the main article, and the waveforms of the downward communication process, as well as the test results at 50kHz, are supplemented here to validate the model proposed in this article. Supplementary Table 2 shows the notations and numbers of the recorded waveforms in various conditions.

Supplementary Table 2 Data transmission waveforms

| Data carrier frequency | Data transmission direction | Data collection location | Waveform number |
|---|---|---|---|
| 100kHz | Downward | $T_x$ CH1, $R_x$ CH3, $R_x$ CH4 | Supplementary Fig. 7 |
| | Downward | $V_i$ CH3, $V_o$ CH4 | |
| 50kHz | Upward | $T_x$ CH1, $R_x$ CH3, $R_x$ CH4 | Supplementary Fig. 8 |
| | Upward | $V_i$ CH4, $V_o$ CH3 | |
| | Downward | $T_x$ CH1, $R_x$ CH3, $R_x$ CH4 | Supplementary Fig. 9 |
| | Downward | $V_i$ CH3, $V_o$ CH4 | |

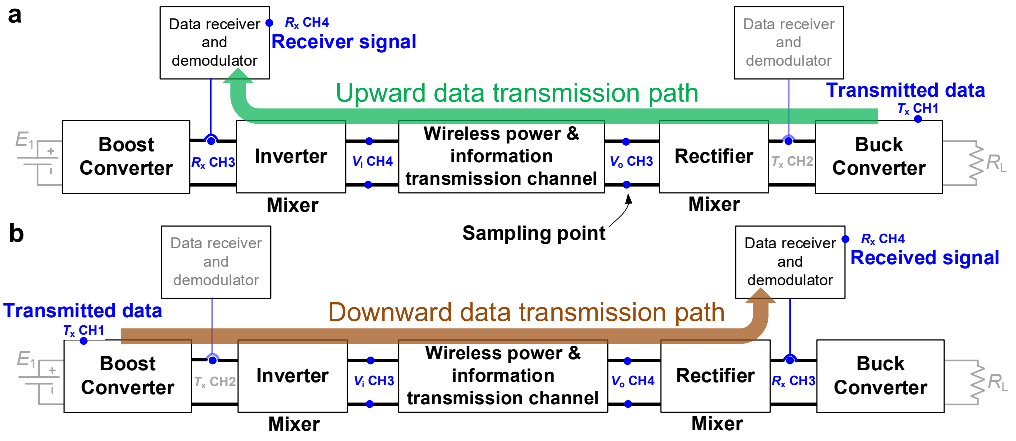


**Supplementary Fig. 6 Waveform notations in the upward and downward communication processes. a,** Green line denotes upward data transmission path, blue line denotes data flow path. **b,** Brown line denotes downward data transmission path.

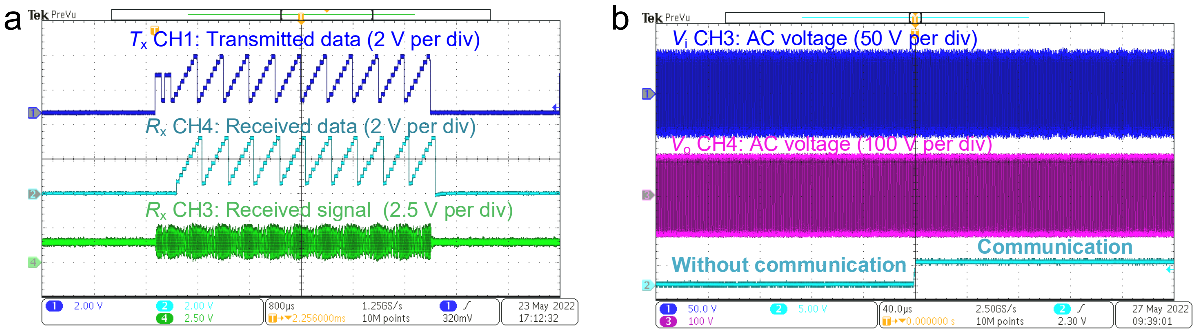


**Supplementary Fig. 7 Waveform in the downward communication processes with data carrier frequency of 100kHz. a,** Transmitted data $T_x$ CH1, received data $R_x$ CH4 and received signal $R_x$ CH3. **b,** AC voltage $V_i$ CH3 and AC voltage $V_o$ CH4.

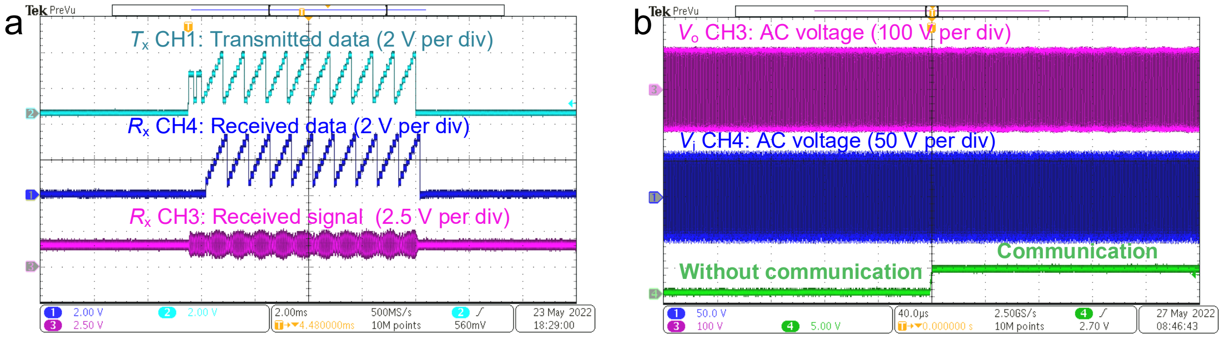


**Supplementary Fig. 8 Waveform in the upward communication processes with data carrier frequency of 50kHz. a,** Transmitted data $T_x$ CH1, received data $R_x$ CH4 and received signal $R_x$ CH3. **b,** AC voltage $V_o$ CH3 and AC voltage $V_i$ CH4.

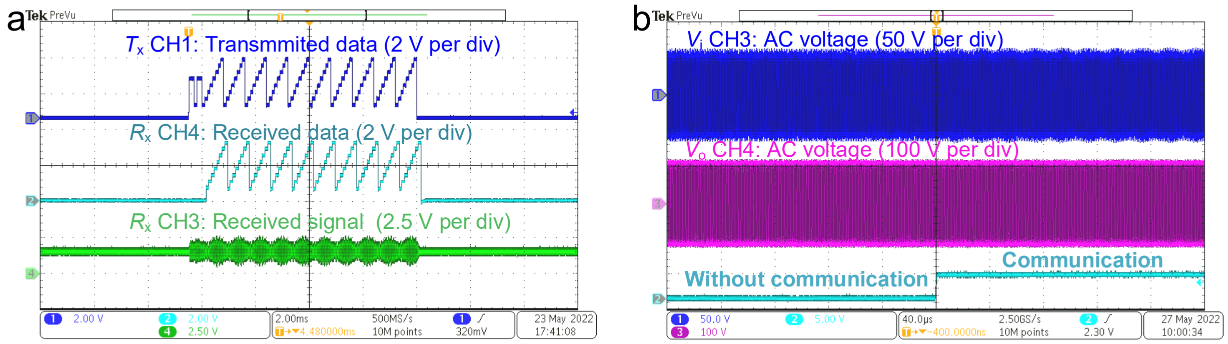


**Supplementary Fig. 9 Waveform in the downward communication processes with data carrier frequency of 50kHz. a,** Transmitted data $T_x$ CH1, received data $R_x$ CH4 and received signal $R_x$ CH3. **b,** AC voltage $V_i$ CH3 and AC voltage $V_o$ CH4.

## Supplementary Discussion 2: Plots of $|S_{21}|$ and $|S_{12}|$

Supplementary Figs. 5e and 5f show the S-parameter sweep of the power and data channels by the vector network analyser (E5061B), and the measurement results are shown in Supplementary Fig. 10.

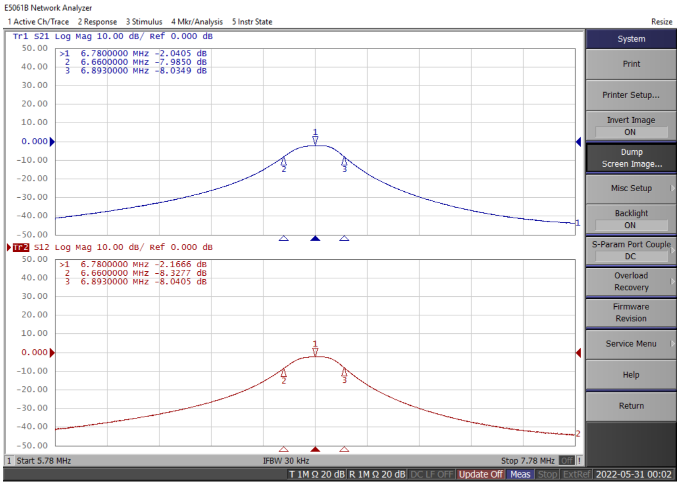


**Supplementary Fig. 10 Plots of $|S_{21}|$ and $|S_{12}|$.**

## Supplementary Discussion 3: Measurement result of upward communication power transfer efficiency

In this experiment, the voltage and current are measured with four high-precision multimeters (FLUCK 289C) and recorded in Supplementary Table 3. The experiment is conducted using the following steps. First, the dc input

voltage on the front side is set to approximately 24V; then, the duty cycle of the buck converter is adjusted based on the load resistance so that the buck's input impedance is set to approximately 320Ω; and finally, the boost converter is regulated to maintain a 48V output voltage on the load side.

Supplementary Table 3 The measurement of the MCR-based WPIDT system (Without and With Communication) during upward communication

| Resistance (Ω) | Input voltage (V) | | Input current (A) | | Duty of Boost | Output voltage (V) | | Output current (A) | | Duty of Buck |
|---|---|---|---|---|---|---|---|---|---|---|
| | Without | With | Without | With | | Without | With | Without | With | |
| 50 | 23.6570 | 23.7230 | 2.9262 | 2.8968 | 0.3650 | 47.5280 | 47.0049 | 0.9480 | 0.9376 | 0.3950 |
| 55 | 23.6810 | 23.7060 | 2.7470 | 2.7374 | 0.3800 | 48.2430 | 47.7199 | 0.8754 | 0.8659 | 0.4100 |
| 60 | 23.7150 | 23.7510 | 2.4982 | 2.4838 | 0.4000 | 47.8570 | 47.3339 | 0.7969 | 0.7882 | 0.4300 |
| 65 | 23.7070 | 23.6890 | 2.2979 | 2.2808 | 0.4150 | 47.5700 | 47.0469 | 0.7316 | 0.7236 | 0.4500 |
| 70 | 23.7510 | 23.7700 | 2.2169 | 2.1090 | 0.4300 | 47.5380 | 47.0149 | 0.6790 | 0.6715 | 0.4650 |
| 75 | 23.7710 | 23.7980 | 2.0083 | 1.9923 | 0.4450 | 47.8150 | 47.2919 | 0.6375 | 0.6305 | 0.4800 |
| 80 | 23.7890 | 23.7250 | 1.8855 | 1.8784 | 0.4600 | 47.8000 | 47.2769 | 0.5981 | 0.5916 | 0.5000 |
| 85 | 23.8030 | 23.7680 | 1.7751 | 1.7611 | 0.4750 | 47.7090 | 47.1859 | 0.5612 | 0.5550 | 0.5150 |
| 90 | 23.8240 | 23.8300 | 1.6686 | 1.6571 | 0.4900 | 47.5670 | 47.0439 | 0.5285 | 0.5227 | 0.5300 |
| 95 | 23.8310 | 23.8220 | 1.5989 | 1.5905 | 0.5000 | 47.7670 | 47.2439 | 0.5030 | 0.4975 | 0.5450 |
| 100 | 23.8390 | 23.8420 | 1.5317 | 1.5235 | 0.5100 | 47.8900 | 47.3669 | 0.4796 | 0.4744 | 0.5600 |

Supplementary Table 4 The measurement power and efficiency of the MCR-based WPIDT system (Without and With Communication) during upward communication

| Resistance (Ω) | Input power (W) | | Output power (W) | | Efficiency | |
|---|---|---|---|---|---|---|
| | Without Com. | With Com. | Without Com. | With Com. | Without Com. | With Com. |
| 50 | 69.2251 | 68.7198 | 45.0565 | 44.0702 | 65.09% | 64.13% |
| 55 | 65.0517 | 64.8920 | 42.2319 | 41.3210 | 64.92% | 63.68% |
| 60 | 59.2448 | 58.9931 | 38.1372 | 37.3081 | 64.37% | 63.24% |
| 65 | 54.4763 | 54.0300 | 34.8022 | 34.0410 | 63.89% | 63.00% |
| 70 | 50.5302 | 50.1300 | 32.2783 | 31.5718 | 63.88% | 62.98% |
| 75 | 47.7393 | 47.4132 | 30.4821 | 29.8188 | 63.85% | 62.89% |
| 80 | 44.8542 | 44.5657 | 28.5892 | 27.9669 | 63.74% | 62.75% |
| 85 | 42.2527 | 41.8567 | 26.7743 | 26.1904 | 63.37% | 62.57% |
| 90 | 39.7527 | 39.4889 | 25.1392 | 24.5893 | 63.24% | 62.27% |
| 95 | 38.1034 | 37.8895 | 24.0268 | 23.5034 | 63.06% | 62.03% |
| 100 | 36.5142 | 36.3235 | 22.9680 | 22.4690 | 62.90% | 61.86% |

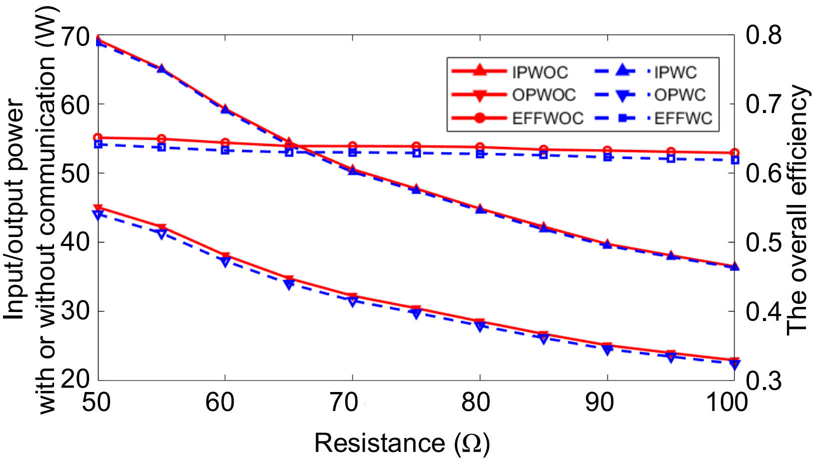


**Supplementary Fig. 11 The overall efficiencies.** Input power without communication (IPWOC). Output power without communication (OPWOC). Input power with communication (IPWC). Output power with communication (OPWC). Efficiency without communication (EFFWOC). Efficiency with communication (EFFWC).

The output efficiency of the cascaded circuits is calculated and listed in Supplementary Table 4, including the results of without and with upward communication under different load resistances. It can be found that the overall efficiency of the system decreases slightly as the output power decreases, but remains above 62%, indicating that the buck converter can maintain the overall efficiency while the boost converter regulates output power. It also shows that the overall efficiency decreased during communication, but only by less than 1%, indicating that the data transmission process had negligible impact on the WPT process. The overall efficiencies are plotted in Supplementary Fig. 11.

**Supplementary Discussion 4: The maximum data rate of WPIDT system**

The maximum data rate is one of the important characteristics in a communication system and there are trade-offs involved if other functions are considered. In this proposed system, the maximal communication rate can be estimated using Shannon's formula $C=B*\log_2(1+S/N)$, assuming the communication channel is an additive white Gaussian noise (AWGN) channel[12].

In our experiment system, the available bandwidth of the channel with the matching circuit is limited to about $B$=40kHz. The signal-to-noise ratio $S/N$ varies with the perturbation depth $\eta$. We conduct an experiment to measure $S/N$ at different values of $\eta$ and the relationship between the maximum data rate $C$ and perturbation depth $\eta$ is depicted in Supplementary Fig. 12. It is observed that $C$ is about 300kbps when $\eta$=0.01 (reasonable in most applications). The data rate is sufficient enough for power management but not compared to Bluetooth or Wi-Fi which need additional chips and circuits.

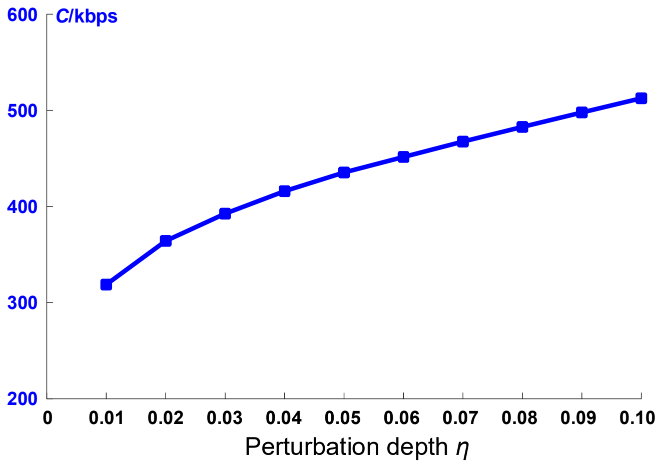


**Supplementary Fig. 12 The relationship between the maximum data rate *C* and perturbation depth *η***
